# Subtilase cytotoxin induces a novel form of Lipocalin 2, which promotes Shiga-toxigenic *Escherichia coli* survival

**DOI:** 10.1038/s41598-020-76027-z

**Published:** 2020-11-03

**Authors:** Kinnosuke Yahiro, Kohei Ogura, Yoshiyuki Goto, Sunao Iyoda, Tatsuya Kobayashi, Hiroki Takeuchi, Makoto Ohnishi, Joel Moss

**Affiliations:** 1 Department of Molecular Infectiology, Graduate School of Medicine, Chiba University, 1-8-1 Inohana, Chuo-ku, Chiba, 260-8670 Japan; 2 Advanced Health Care Science Research Unit, Institute for Frontier Science Initiative, Kanazawa University, Kanazawa, 920-0942 Japan; 3 Division of Molecular Immunology, Medical Mycology Research Center, Chiba University, Chiba, 260-8670 Japan; 4 Division of Mucosal Symbiosis, International Research and Development Center for Mucosal Vaccines, Institute of Medical Science, The University of Tokyo, Tokyo, 108-8639 Japan; 5 AMED-PRIME, Japan Agency for Medical Research and Development, Tokyo, 100-0004 Japan; 6 Department of Bacteriology I, National Institute of Infectious Diseases, Tokyo, 162-8640 Japan; 7 Reproductive Medicine, Graduate School of Medicine, Chiba University, Chiba, 260-8670 Japan; 8 Clinical Laboratory, University of Tsukuba Hospital, Tsukuba, 305-8576 Japan; 9 Pulmonary Branch, National Heart, Lung, and Blood Institute, National Institutes of Health, Bethesda, MD 20892-1590 USA

**Keywords:** Cell death, Cell signalling, Glycobiology, Mechanisms of disease, Antimicrobials, Bacteria, Cellular microbiology, Pathogens, Cell biology, Microbiology, Molecular biology, Infectious diseases, Bacterial infection

## Abstract

Shiga-toxigenic *Escherichia coli* (STEC) infection causes severe bloody diarrhea, renal failure, and hemolytic uremic syndrome. Recent studies showed global increases in Locus for Enterocyte Effacement (LEE)-negative STEC infection. Some LEE-negative STEC produce Subtilase cytotoxin (SubAB), which cleaves endoplasmic reticulum (ER) chaperone protein BiP, inducing ER stress and apoptotic cell death. In this study, we report that SubAB induces expression of a novel form of Lipocalin-2 (LCN2), and describe its biological activity and effects on apoptotic cell death. SubAB induced expression of a novel LCN2, which was regulated by PRKR-like endoplasmic reticulum kinase via the C/EBP homologous protein pathway. SubAB-induced novel-sized LCN2 was not secreted into the culture supernatant. Increased intracellular iron level by addition of holo-transferrin or FeCl_3_ suppressed SubAB-induced PARP cleavage. Normal-sized FLAG-tagged LCN2 suppressed STEC growth, but this effect was not seen in the presence of SubAB- or tunicamycin-induced unglycosylated FLAG-tagged LCN2. Our study demonstrates that SubAB-induced novel-sized LCN2 does not have anti-STEC activity, suggesting that SubAB plays a crucial role in the survival of LEE-negative STEC as well as inducing apoptosis of the host cells.

## Introduction

Shiga-toxigenic *Escherichia coli* (STEC) is a food-borne pathogen, which causes bloody diarrhea, renal failure, and hemolytic-uremic syndrome (HUS)^1^. Serotype O157:H7 is the major strain found in STEC infection, and produces Shiga toxin (Stx) 1 and/or Stx2, which are virulence factors associated with severe gastrointestinal disease^2^. Other serotypes of STEC or a hybrid strain, Enteroaggregative *E. coli* (EAEC)/STEC, were also associated with disease outbreaks in Germany^3^, Argentina^4^, and Sweden^5^. In addition, Locus for Enterocyte Effacement (LEE)-negative STEC infection has shown a global increase^6^. STEC O113:H21 98KN2 strain was associated with an outbreak of HUS in Australia. This LEE-negative STEC strain produced two cytotoxins, Stx2 and subtilase cytotoxin (SubAB)^7^.

SubAB is a member of the family of AB_5_ cytotoxins, which consists of a subtilase-like A subunit (35-kDa) and pentamer of receptor recognition domain B subunits (15-kDa)^7^. Initially, SubAB binds to sialic acid-modified, cell-surface receptors^8–10^, and enters into cells via clathrin-mediated^11^ and lipid raft- and actin-dependent pathways^12^. In the endoplasmic reticulum (ER), SubAB cleaves a specific site on the chaperone protein BiP/Grp78^7^, which leads to activation of ER stress-sensor proteins (e.g., IRE1, ATF6, PERK)^13,14^. Activated stress signaling induces a variety of cell responses (e.g., inhibition of protein synthesis, cell cycle arrest, apoptosis, inhibition of iNOS synthesis, stress granule formation)^14–21^. SubAB-induced apoptosis in HeLa cells was suppressed by steroids or diacylglycerol analogues^22^. However, these inhibitors did not suppress SubAB-induced lethal severe hemorrhagic inflammation in mice^22^.

In response to bacterial invasion, mammalian cells secrete a variety of antimicrobial agents such as antimicrobial peptides (AMPs)^23^. In mammalian cells, the two major AMP families are the cathelicidins and defensins, which are composed of 10–50 amino acid residues. Cathelicidins and defensins bind directly to bacterial membranes, inducing membrane damage and death^24^. Besides these AMPs, mammalian cells inhibit bacterial growth by producing Lipocalin-2 (LCN2), a secretary glycoprotein that binds siderophores and prevents delivery of iron to the bacteria^25^. In various cells and tissues, LCN2 expression was induced by a variety of factors (e.g., lipopolysaccharide, cytokines, retinoic acids, growth factors, insulin)^26^ and regulated transcription factors such as nuclear factor-kB (NF-kB), C/EBP, and STAT1^27,28^. The *lcn2*-deficient mice showed increased sensitivity to some siderophore-producing bacterial infections^29–32^. Increased LCN2 expression was observed in the metabolic inflammation of obesity^33^ and in the intestine of inflammatory bowel diseases^34^. LCN2 played a protective effect against intestinal inflammation and tumorigenesis associated with alterations of the microbiota^35^. In addition to its bacteriostatic activity, LCN2 also acted as a biomarker of inflammation, ischemia, infection and kidney injury^36^. ER stress-mediated signaling pathways also promoted upregulation of LCN2 expression^37,38^.

The aim of this study was to identify the effects of SubAB on LCN2 expression and biological activity. We found that SubAB-caused ER stress induced a novel size of LCN2 through ER stress sensor protein, PERK. Knockdown of C/EBP homology protein (CHOP), an ER-stress inducible protein and transcription factor, inhibited SubAB-induced LCN2 production. LCN2 knockdown by siRNA was promoted SubAB-induced apoptosis, while overexpression of FLAG-tagged LCN2 did not affect SubAB-induced PARP cleavage. In support of the importance of iron in the pathway, SubAB-induced PARP cleavage was suppressed in the presence of holo-transferrin and FeCl_3_. Further, the growth of STEC O157:H7 Sakai strain or O113:H21 strain was suppressed by incubation with normal LCN2, which did not induce production of the toxins by the bacteria. Interestingly, the molecular size of LCN2 induced by SubAB was different from that seen following treatment with tunicamycin, poorly detected in the culture supernatant, and exhibited attenuated antimicrobial activity compared to control. The amino acid sequences of the novel and standard LCN2 were identical, consistent with altered post-translational modification of the novel protein. Thus, our findings demonstrate that SubAB induced a novel non-secreted form of LCN2, which would promote survival of LEE-negative STEC.

## Results

### SubAB induces lipocalin 2 (LCN2) expression

**Figure 1:**
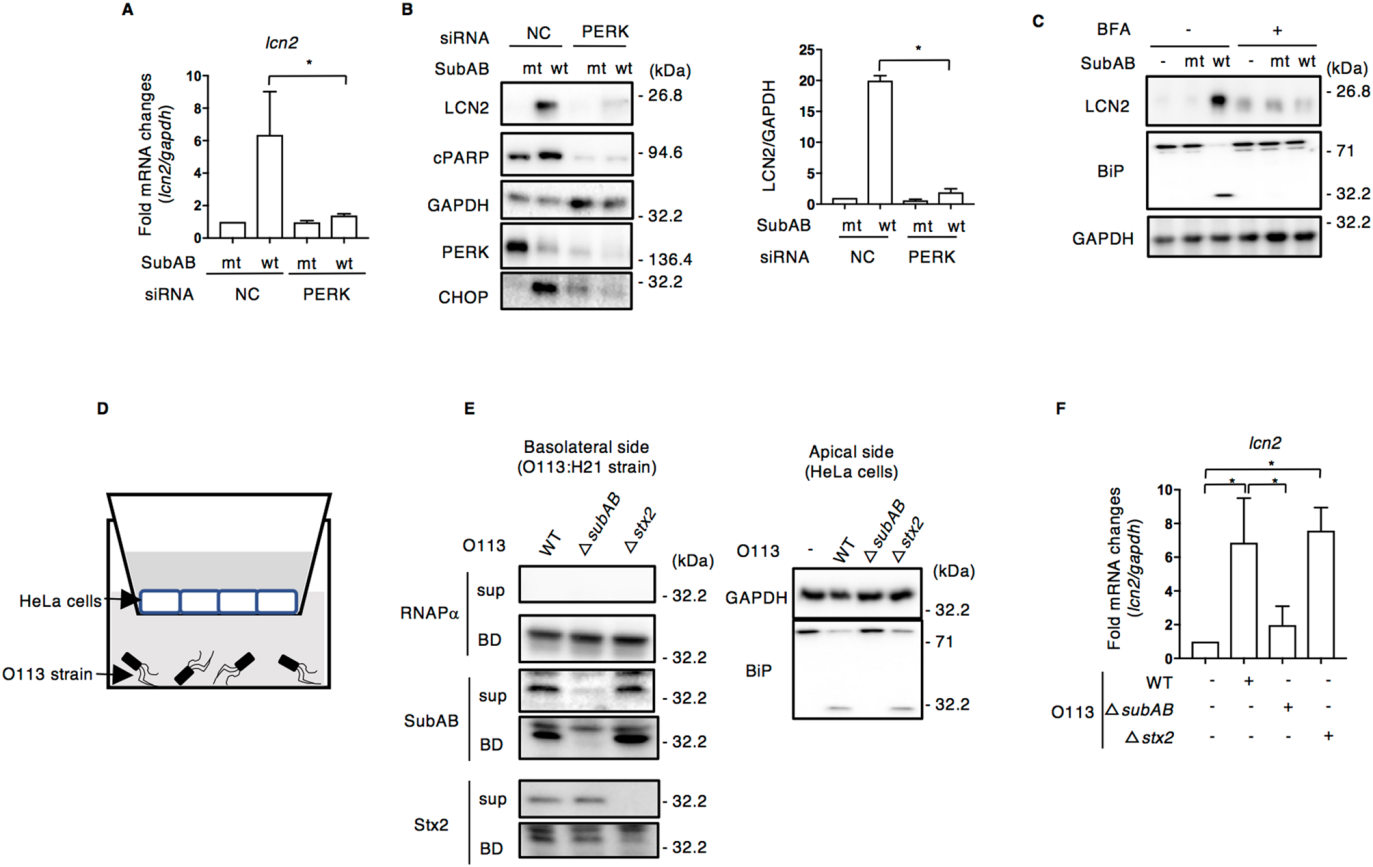
SubAB induces LCN2 expression. (**A**) Control (NC) or PERK siRNA-transfected HeLa cells were incubated for 24 h with 400 ng ml^−1^ of catalytically inactive SubA_S272A_B (mt) or SubAB (wt). The mRNA levels of *lcn2* was measured by RT-qPCR as described in “Methods”. GAPDH was used as an internal control. Data are mean ± SD (n = 3). *P < 0.05, versus mt SubAB-treated control cells. (**B**) Cell lysates were subjected to immunoblotting with the indicated antibodies. GAPDH served as a loading control. Quantification of LCN2 in HeLa cells was performed by densitometry. Data are presented as mean ± SD of values from three independent experiments and significance is *P < 0.05. (**C**) HeLa cells were preincubated for 30 min with 10 mM BFA, and then incubated for 24 h with control PBS (−), 400 ng ml^−1^ of mt or wt SubAB in the presence or absence of the BFA. Cell lysates were subjected to immunoblotting with the indicated antibodies. GAPDH served as a loading control. (**D**) Schematic drawing of co-culture system. Confluent HeLa cells were plated on apical side (Apical), which has a semipermeable membrane. The wild-type, *∆subAB*, or *∆stx2* STEC O113:H21 strain (1–2.5 × 10^5^ cfu) was plated on the basolateral side (Baso) and the system cultured for 24 h. (**E**) HeLa cells were lysed with 1xSDS sample buffer for immunoblotting with the indicated antibodies. After centrifugation of STEC culture medium on the basolateral side, bacterial body (BD) or culture supernatant (sup) was collected and then lysed with 1xSDS sample buffer for immunoblotting with the indicated antibodies. GAPDH or RNAPα was used as an internal control. (**F**) HeLa cells were co-cultured for 24 h with the indicated STEC strains as shown in (**D**). The *lcn2* mRNA levels were measured by RT-qPCR as described in “Methods”. GAPDH was used as an internal control. Data are mean ± SD (n = 3). *P < 0.05, versus mt SubAB-treated control cells.

To understand the effect of STEC O113:H21-produced SubAB on defense factors generated by host cells, we focused on LCN2, a protein that was induced by ER stress^38^ and acts as an anti-microbial defense factor by binding to a subset of bacterial siderophores^25^. In this study, we primarily used HeLa cells mainly as in our previous our reports^10,12,14,22^. Both Caco2 cells and HCT116 cells were difficult to analyze for SubAB-induced apoptosis. To assess the influence of SubAB on the production of LCN2 in HeLa cells, we used real-time quantitative reverse transcriptase polymerase chain reaction (RT-qPCR) and Western blotting. Transcription of *lcn2* mRNA was significantly increased by purified wild-type (wt) SubAB compared to catalytically inactivated mutant (mt) SubAB. PERK (RNA-dependent protein kinase (PKR)-like ER kinase), a key ER stress sensor of the unfolded protein response, is responsible for SubAB-induced apoptosis^14^. SubAB-increased *lcn2* mRNA expression was suppressed in PERK-knockdown cells (Fig. 1A).

We detected wt SubAB-enhanced PARP cleavage (cPARP), and CHOP and LCN2 protein expression, which were not seen in PERK-knockdown cells (Fig. 1B). Further, to show that BiP cleavage by SubAB is essential for LCN2 expression, we investigated the effect of Brefeldin A (BFA), which inhibits retrograde transport by disrupting the Golgi apparatus, on SubAB-increased LCN2. BFA is known to inhibit SubAB-mediated BiP cleavage^7^. HeLa cells were pretreated with or without 10 mM BFA for 30 min, and then SubAB was added to cells. After a 24 h incubation, LCN2 expression was detected by Western blotting analysis (Fig. 1C). SubAB-increased LCN2 was significantly suppressed in the presence of BFA. BFA alone slightly increased low molecular weight of LCN2 expression compared to SubAB-increased LCN2, suggesting that BFA may affect ER stress signaling pathway by disrupting the Golgi apparatus.

We next investigated whether STEC O113:H21-secreted SubAB participated in the regulation of LCN2 expression using a co-culture system (Fig. 1D). To show that SubAB is a critical inducer of LCN2, HeLa cells were incubated with three types of STEC O113:H21 (e.g., wild-type, *subAB*-knockout, *stx2*-knockout). After a 24 h co-culture of HeLa cells with STEC O113:H21 strains, bacteria were collected by centrifugation; expression of SubAB or Stx2 was assessed in bacteria and culture supernatant by Western blotting analysis. As shown in Fig. 1E, SubAB and Stx2 were detected in wild-type O113:H21 bacteria and culture supernatant. In *subAB*-deficient O113:H21 strain, as expected, SubAB was not detected, but Stx2 was clearly observed in bacteria and its culture supernatant. SubAB was detected, but not Stx2 in *stx2*-deficient O113:H21 bacteria and its culture supernatant. Consistent with SubAB expression, SubAB-induced BiP cleavage was detected in co-culture with HeLa cells. *lcn2* mRNA expression was significantly increased in wild-type and *stx2*-deficient O113:H21 co-cultured HeLa cells, but not in the *subAB*-deficient strain (Fig. 1F). These findings suggest that SubAB induces LCN2 expression via BiP cleavage, followed by PERK signaling.

### Transcription factor C/EBP homologous protein (CHOP) is involved in SubAB-induced LCN2 expression

**Figure 2:**
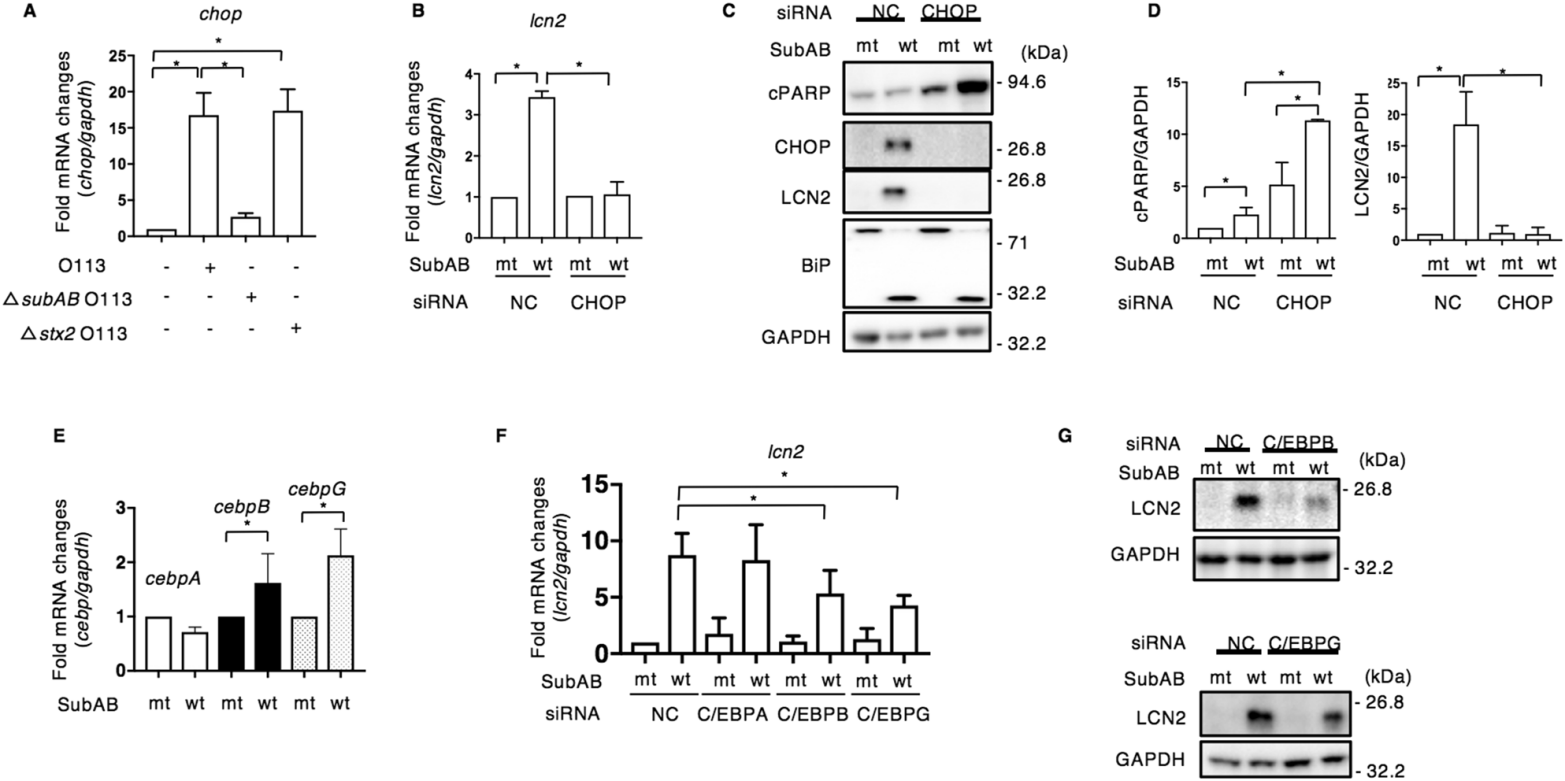
SubAB-increased CHOP and C/EBPG regulate SubAB-induced LCN2 expression. (**A**) HeLa cells were co-cultured for 24 h with the indicated STEC O113:H21 strains as shown in Fig. 1D. The *chop* mRNA levels were measured by RT-qPCR as described in “Methods”. GAPDH was used as an internal control. Data are mean ± SD (n = 3). *P < 0.05, versus mt SubAB-treated control cells. (**B**) Control (NC) or CHOP siRNA-transfected cells were incubated for 24 h with mt SubAB or SubAB (400 ng ml^−1^). The *lcn2* mRNA levels were measured by RT-qPCR as described in “Methods”. GAPDH was used as an internal control. Data are mean ± SD (n = 3). *P < 0.05, versus mt SubAB-treated control cells. (**C**) Control (NC) or CHOP siRNA-transfected cells were incubated for 24 h with mt or wt SubAB (400 ng ml^−1^). Cell lysates were subjected to immunoblotting with the indicated antibodies. GAPDH served as a loading control. (**D**) Quantification of cPARP or LCN2 in the transfected cells with mt or wt SubAB was performed by densitometry. Data are presented as mean ± SD of values from three independent experiments and significance is *P < 0.05. (**E**) Cells were incubated for 24 h with mt or wt SubAB (400 ng ml^−1^). The mRNA levels of *cebpA*, *cebpB*, or *cebpG* were measured by RT-qPCR as described in “Methods”. GAPDH was used as an internal control. Data are mean ± SD (n = 3). * *P* < 0.05, versus mt SubAB-treated control cells. (**F**) The indicated siRNA-transfected cells were incubated for 24 h with mt or wt SubAB (400 ng ml^−1^). The *lcn2* mRNA levels were measured by RT-qPCR as described in “Methods”. GAPDH was used as an internal control. Data are mean ± SD (n = 3). *P < 0.05, versus mt SubAB-treated control cells. (**G**) Control (NC), C/EBPB, or C/EBPG siRNA-transfected cells were incubated for 24 h with mt or wt SubAB (400 ng ml^−1^). Cell lysates were subjected to immunoblotting with anti-LCN2 antibodies. GAPDH served as a loading control.

CHOP, which is known as an ER-stress marker, plays an essential role in cell cycle arrest and apoptosis, and also acts as a transcriptional regulator^39^. A previous study reported that LCN2 is a CHOP target gene that mediates ER stress-induced apoptosis in A549 cells^40^. We investigated whether SubAB-increased CHOP is involved in LCN2 expression in HeLa cells. When cells were co-cultured with the three types of O113:H21 strains as shown in Fig. 1D, the level of *chop* mRNA was increased in wild-type and *stx2*-deficient O113:H21 co-cultured with HeLa cells (Fig. 2A). Further, we examined whether CHOP is a crucial inducer of LCN2 expression; CHOP-knockdown cells were incubated for 24 h with mt or wt SubAB. We found that wt SubAB-induced *lcn2* mRNA expression was inhibited in CHOP-knockdown cells (Fig. 2B, Supplementary Fig. S1). In agreement with mRNA expression, we detected that SubAB-stimulated CHOP and LCN2 proteins were suppressed, while SubAB-induced PARP cleavage (cPARP) was promoted, in CHOP-knockdown cells (Fig. 2C,D). It was reported that LCN2 expression by thapsigargin was regulated by both CHOP and C/EBPB^40^. Our microarray analysis data of wt SubAB-treated cells showed that mRNAs of *cebpB* (ratio, 3.31) and *cebpG* (ratio, 3.78) were increased compared to mt SubAB-treated cells. The mRNA levels of both *cebpB* and *cebpG* were significantly increased after incubation with wt SubAB for 24 h (Fig. 2E). We next investigated if C/EBPB or C/EBPG are involved in wt SubAB-stimulated LCN2 expression by using specific siRNA-transfected cells. First, we confirmed that C/EBPB or C/EBPG siRNA suppressed the expression of their respective mRNA (Supplementary Fig. S2A). SubAB-stimulated *lcn2* mRNA was decreased in C/EBPB and C/EBPG siRNA-transfected cells, but not in cells transfected with C/EBPA siRNA (Fig. 2F). Consistent with the mRNA expression results, SubAB-stimulated LCN2 protein was reduced in C/EBPB- and/CEBPG-knockdown cells compared to control cells (Fig. 2G). Interestingly, SubAB-stimulated *chop* mRNA was suppressed in both C/EBPB- and C/EBPG-knockdown cells (Supplementary Fig. S2B).

**Figure 3:**
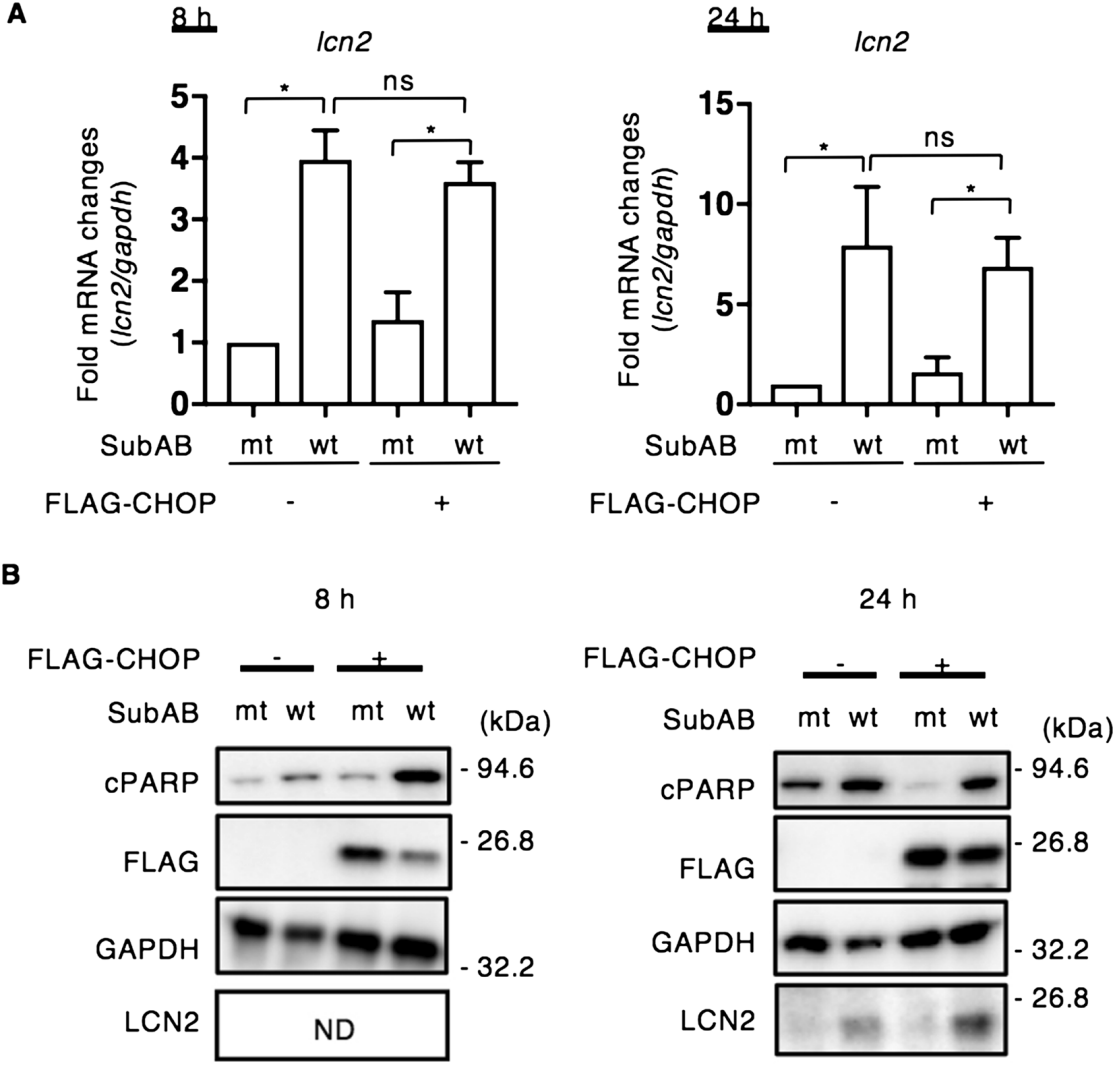
Effect of CHOP overexpression on LCN2 expression by SubAB. (**A**) Control or FLAG-tagged CHOP plasmid-transfected HeLa cells were incubated for 24 h with mt SubAB or SubAB (400 ng ml^−1^). The *lcn2* mRNA levels were measured by RT-qPCR as described in “Methods”. GAPDH was used as an internal control. Data are mean ± SD (n = 3). *P < 0.05, versus mt SubAB-treated control cells. *Ns*: not significant. (**B**) Cell lysates from 8 h or 24 h incubation with toxins were subjected to immunoblotting with the indicated antibodies. GAPDH served as a loading control. Experiments were repeated three times with similar results. *ND*: not detected.

Overexpression of CHOP has been demonstrated to cause cell cycle arrest and/or apoptosis^41^. We next examined the effect of overexpressed CHOP on SubAB-induced LCN2. Cells transfected with FLAG-tagged CHOP expression plasmid were incubated for 24 h with mt or wt SubAB. SubAB-stimulated *lcn2* mRNA was not altered in CHOP-overexpressing cells (Fig. 3A). We also tested whether SubAB-stimulated CHOP and overexpressed CHOP localized to the nucleus. CHOP was mainly localized in the nucleus (Supplementary Fig. S3). In CHOP-overexpressing cells, SubAB-induced PARP cleavage was significantly increased after 8 h incubation, but LCN2 was not detected. After a 24 h incubation, SubAB-induced PARP cleavage and LCN2 expression were found at similar levels in control and CHOP-expressing cells (Fig. 3B). These findings suggest that SubAB-induced LCN2 expression is mainly regulated by CHOP, which is controlled by C/EBPB and C/EBPG. CHOP expression alone did not induce LCN2 protein. Thus, LCN2 expression required not only CHOP but also activation of ER stress signaling.

### LCN2 participates in SubAB-induced apoptosis

**Figure 4:**
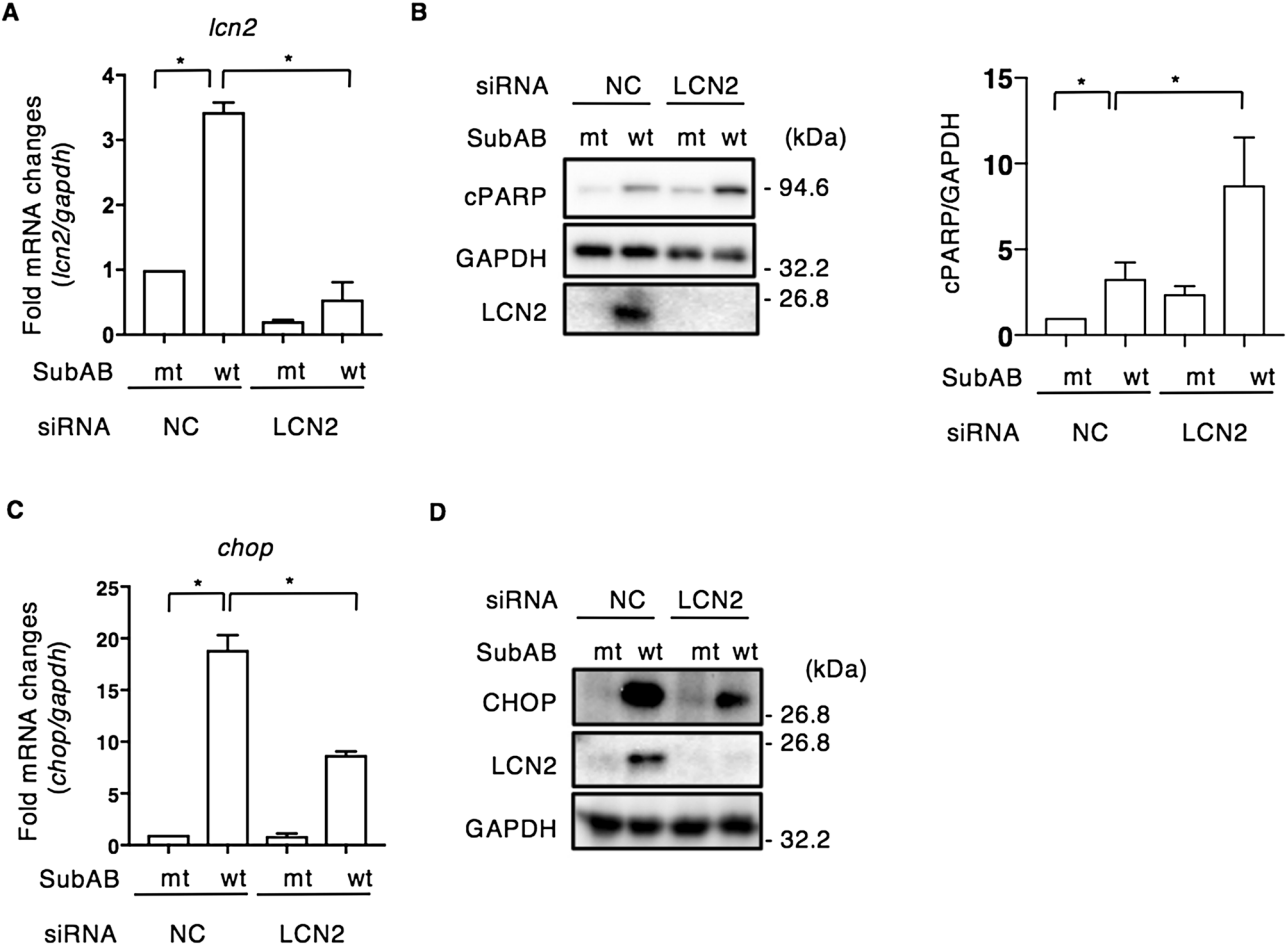
Knockdown of LCN2 increases SubAB-induced apoptosis. (**A**) Control (NC) or LCN2 siRNA-transfected cells were incubated for 24 h with mt SubAB or SubAB (400 ng ml^−1^). The *lcn2* mRNA levels were measured by RT-qPCR as described in “Methods”. GAPDH was used as an internal control. Data are mean ± SD (n = 3). *P < 0.05, versus mt SubAB treated control cells. (**B**) Cell lysates were subjected to immunoblotting with the indicated antibodies. GAPDH served as a loading control. Quantification of cPARP in HeLa cells was performed by densitometry (right panel). Data are presented as mean ± SD of values from three independent experiments and significance is *P < 0.05. (**C**) Control (NC) or LCN2 siRNA-transfected cells were incubated for 24 h with mt or wt SubAB (400 ng ml^−1^). The *chop* mRNA levels were measured by RT-qPCR as described in “Methods”. GAPDH was used as an internal control. Data are mean ± SD (n = 3). *P < 0.05, versus mt SubAB-treated control cells. (**D**) Cell lysates were subjected to immunoblotting with the indicated antibodies. GAPDH served as a loading control. Experiments were repeated three times with similar results.

Several studies have shown that LCN2 plays a crucial role as a protector or enhancer of stress-induced apoptosis in various tissues or cells^42–45^. We investigated next whether LCN2 participated in SubAB-induced apoptosis. We assessed the efficiency of LCN2 siRNA by RT-qPCR. *lcn2* mRNA expression was not increased by SubAB in LCN2-knockdown cells. The catalytically inactive SubAB (mt SubAB) had no effect (Fig. 4A). We investigated if knockdown of LCN2 by siRNA affected SubAB-induced apoptosis. In LCN2-knockdown cells, SubAB-induced LCN2 expression was suppressed, and PARP cleavage, a hallmark of apoptosis, was significantly enhanced compared to control siRNA-transfected cells (Fig. 4B). Previous studies reported that LCN2 attenuated NF-κB subunit p65 activation under hypoxic conditions^46^ and regulated macrophage polarization and activation of NF-kB/STAT3 signaling^47^. We next investigated the effect of LCN2 knockdown on *chop* mRNA expression by RT-qPCR. SubAB-induced *chop* mRNA expression was decreased in LCN2-knockdown cells (Fig. 4C). Consistent with the mRNA expression, SubAB-induced CHOP was significantly attenuated in LCN2-knockdown cells by Western blot analysis (Fig. 4D).

**Figure 5:**
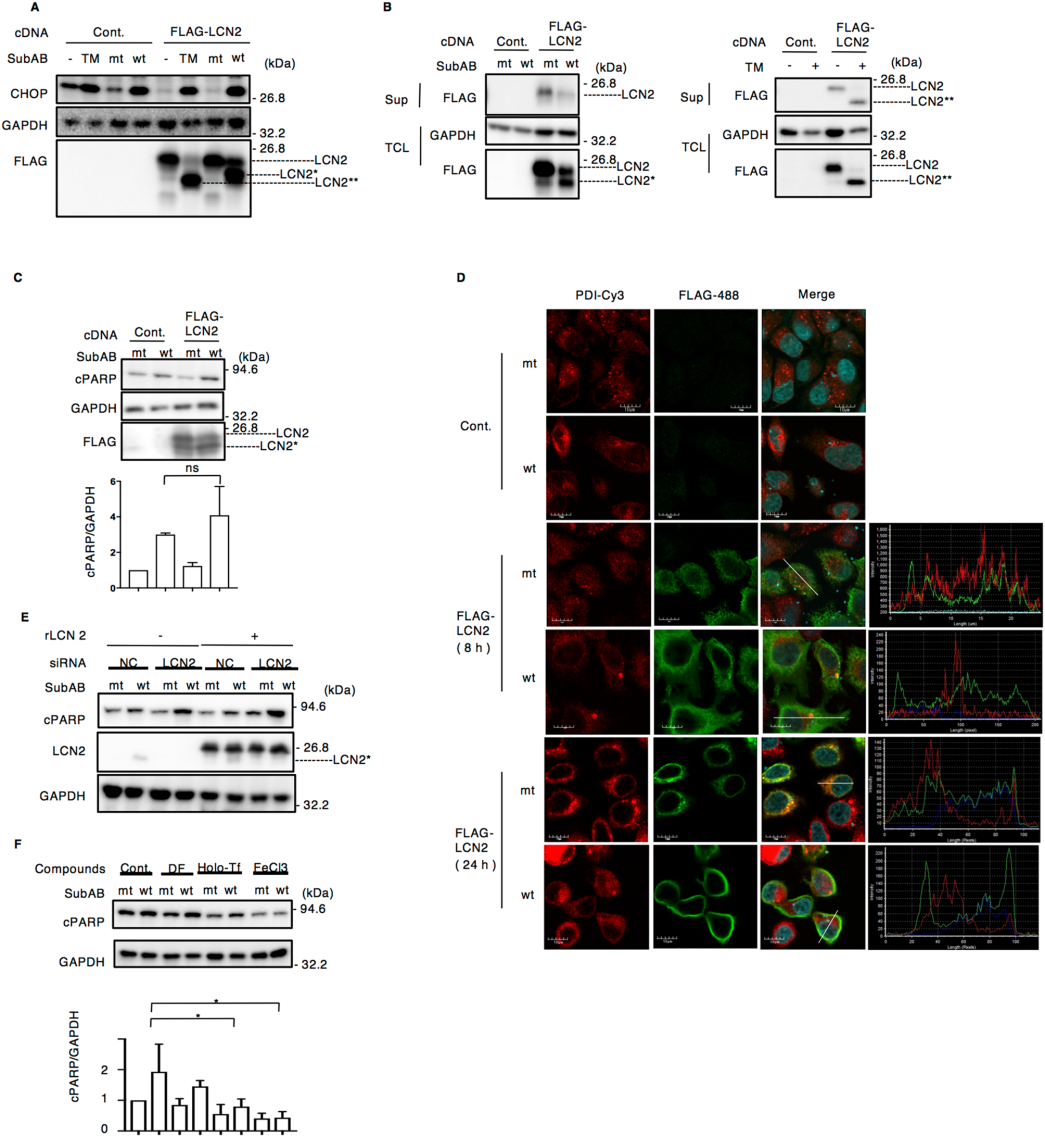
SubAB induces novel form of LCN2. (**A**) Control and FLAG-tagged LCN2 plasmid-transfected HeLa cells were incubated for 24 h with tunicamycin (TM, 1 μg ml^−1^), mt or wt SubAB (400 ng ml^−1^). Cell lysates were subjected to immunoblotting with the anti-CHOP and anti-FLAG antibodies. GAPDH served as a loading control. Experiments were repeated three times with similar results. (**B**) The indicated cDNA-transfected cells were incubated for 24 h with mt SubAB, wt SubAB or TM. Cell lysates (TCL) or culture supernatant (Sup) was subjected to immunoblotting with the anti-FLAG antibodies. GAPDH served as a loading control. Experiments were repeated three times with similar results. (**C**) The indicated cDNA-transfected cells were incubated for 24 h with mt SubAB or wt SubAB. Cell lysates were subjected to immunoblotting with the anti-FLAG and anti-cPARP antibodies. GAPDH served as a loading control. Data are presented as mean ± SD of values from three independent experiments and significance is *P < 0.05. (**D**) The indicated cDNA-transfected cells were incubated for 8 h or 24 h with mt or wt SubAB (400 ng ml^−1^). Cells were fixed with 4% PFA and reacted with the anti-PDI (red) or anti-FLAG antibodies (green) and observed by confocal microscopy. Cell nuclei were stained by DAPI (cyan). Fluorescence intensity was quantified by the white bar in the picture by FV10i-LIV analysis software. (**E**) Control (NC) or LCN2 siRNA-transfected cells were incubated for 24 h with mt or wt SubAB in the presence of purified rLCN2 (1.5 μg per well). Cell lysates were subjected to immunoblotting with anti-cPARP and anti-LCN2 antibodies. GAPDH served as a loading control. Experiments were repeated three times with similar results. (**F**) Cells were incubated for 12 h with mt or wt SubAB (400 ng ml^−1^) in the presence of 100 μM DF, 100 μg ml^−1^ holo-Tf, or 50 μM FeCl_3_. Cell lysates were subjected to immunoblotting with anti-cPARP antibodies. GAPDH served as a loading control. Experiments were repeated three times with similar results. Data are presented as mean ± SD of values from three independent experiments and significance is *P < 0.05.

Next, we tested the effect of LCN2 overexpression on SubAB-induced CHOP expression (Fig. 5A). We used tunicamycin (TM), a chemical ER stress inducer and *N*-glycosylation inhibitor, as a positive control^48^. After transfection for 48 h with control or FLAG-tagged LCN2 plasmid, cells were incubated for 24 h with TM, or mt or wt SubAB. CHOP expression by TM or wt SubAB was not different between control and FLAG-tagged LCN2-transfected cells. Interestingly, we observed three different molecular sizes of LCN2 in the presence of TM or wt SubAB in FLAG-tagged LCN2-overexpressing cells. Thus, TM caused a shift to a lower molecular weight form of unglycosylated LCN2 (LCN2**), while SubAB-induced LCN2 was observed in two bands, which consist of normal size and a slightly lower molecular size LCN2 (LCN2*). Since LCN2 is a secreted protein^49^, we investigated whether the stimulated LCN2 was secreted into the culture supernatant. Although the normal size of FLAG-tagged LCN2 was detected in the culture supernatant, SubAB-induced low molecular weight LCN2 (LCN2*) was not observed. Of note, TM-induced FLAG-tagged unglycosylated LCN2 (LCN2**) was detected in the culture supernatant (Fig. 5B). Since overexpression of LCN2 was protective from inflammation-associated cell death by LPS^50^, we next examined the effect of LCN2 overexpression on SubAB-induced apoptosis (Fig. 5C). In FLAG-tagged LCN2 transiently transfected cells, SubAB-induced PARP cleavage in control cells was not significantly different from that seen in LCN2-overexpressing cells after a 24 h incubation. We next investigated the localization of SubAB-increased novel FLAG-LCN2 by immunostaining. In mt SubAB-treated cells, FLAG-tagged LCN2 was observed in the cytosolic compartment and partially co-localized with ER marker, protein disulfide isomerase (PDI). However, SubAB-induced novel LCN2 (LCN2*) was also mainly localized in the cytosolic compartment after an 8 h incubation, and distributed at cell membrane after a 24 h incubation (Fig. 5D, Supplementary Fig. S4). We tested the protective effects of extracellular LCN2 on SubAB-induced cell death. Cells were transfected with siRNA for LCN2, and then incubated for 24 h with SubAB in the presence or absence of purified recombinant human LCN2 (rLCN2). SubAB-induced PARP cleavage was enhanced in LCN2-knockdown cells, which was not altered even in the presence of purified LCN2 (Fig. 5E). These results suggest that suppression of LCN2 or excess presence of rLCN2 in cells promotes an ER stress-induced apoptotic pathway. In addition, SubAB-increased novel LCN2 (LCN2*), was not secreted into culture supernatant, but was accumulated at the cell membrane. Our next question is whether intracellular iron level affects SubAB-induced cell death signaling. We tested whether SubAB-induced PARP cleavage was prevented by delivery of iron by using holo-transferrin (holo-Tf) or ferric chloride (FeCl_3_). The results of Fig. 5F show that addition of either holo-Tf or FeCl_3_ suppressed SubAB-induced PARP cleavage. The iron chelator deferoxamine (DF) had no effect on SubAB-induced PARP cleavage after 12 h incubation. These findings suggest that the SubAB-stimulated apoptotic pathway was suppressed by increased intracellular iron concentration.

### LCN2 suppressed STEC O113:H21 growth

**Figure 6:**
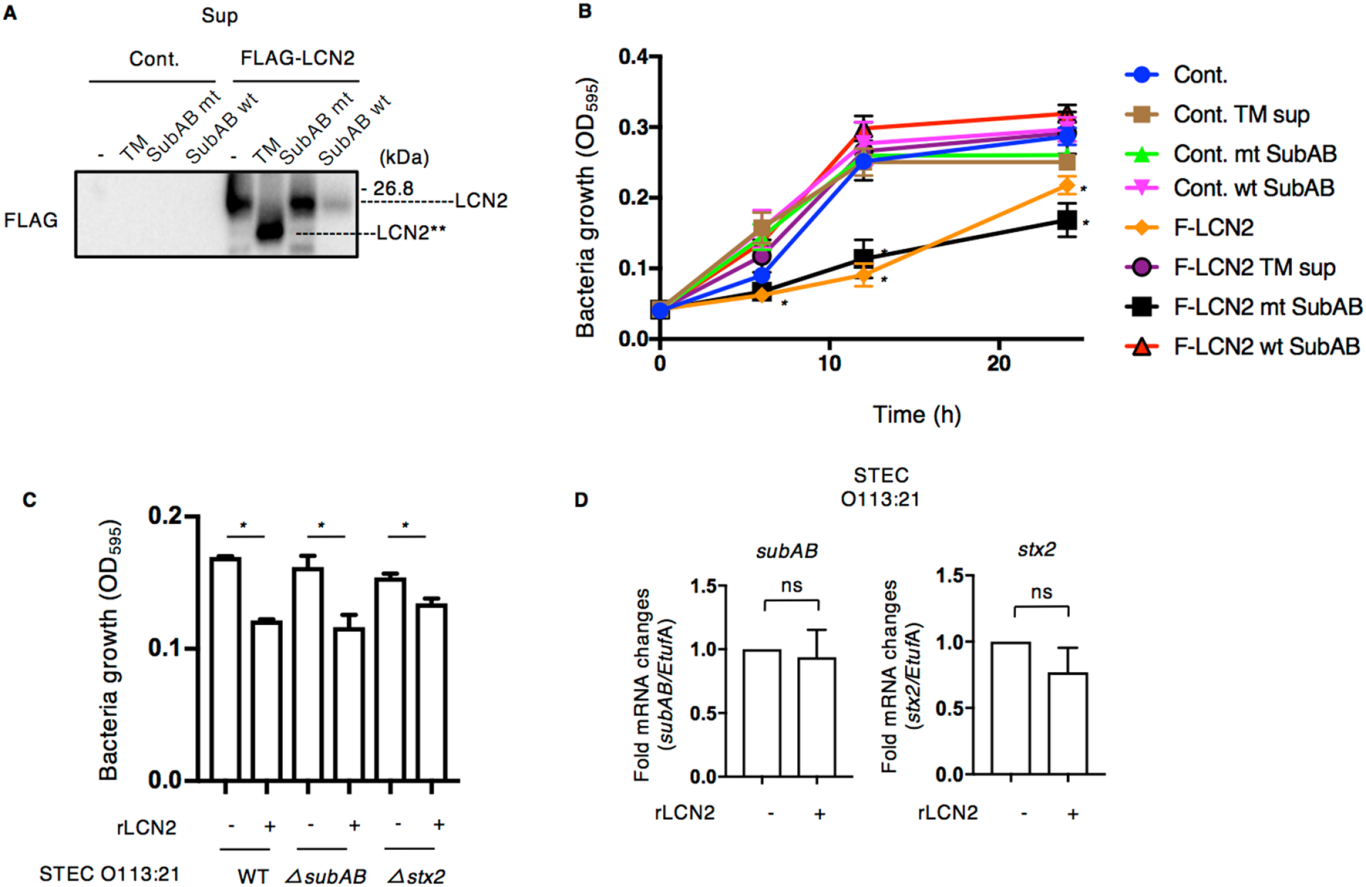
The growth of STEC O113:H21 is decreased by LCN2. (**A**) Culture supernatants (Sup) from control DMSO, 1 μg ml^−1^ TM, mt SubAB or wt SubAB incubated for 24 h with control or FLAG-tagged LCN2 (F-LCN2) transfected cells. The Sup proteins were analyzed by Western blotting using anti-FLAG antibodies. The blots shown are representative of three independent experiments. (**B**) STEC O113:H21(1–2.5 × 10^3^ cfu) were incubated with these culture supernatants and then bacteria growth was monitored by OD_595_ at 0, 6, 12, and 24 h. Experiments were repeated two times with similar results. (**C**) STEC O113:H21 wild-type, *ΔsubAB orΔstx2* strains were grown in LB broth overnight, diluted with RPMI1640 medium (1–2.5 × 10^3^ cfu/100 μl) with or without 0.5 μg purified recombinant human LCN2 (rLCN2). After 18 h incubation at 37 °C, bacteria growth was measured by OD_595_. (**D**) Culture medium with or without rLCN2 was centrifuged and STEC O113:H21 wild-type strains were collected. The levels of *subAB* and *stx2* mRNA were analyzed by RT-qPCR. *Etuf*A was used as an internal control.

LCN2 secreted from host cells captures bacterial siderophores and inhibits iron reuptake into bacteria^51^. Further, LCN2 inhibited growth of a nonpathogenic clinical isolate of *E. coli* and protected mammals from *E. coli* infection^29,52,53^. Here, we investigated the effect of ER stress-induced LCN2 on growth of STEC O113:H21, a pathogenic clinical isolate. We collected the supernatants from control or FLAG-tagged LCN2-transfected cells in the presence or absence of SubAB or TM, incubated STEC O113:H21 with supernatants, and then measured bacterial growth at the indicated time points. At 24 h incubation, cultured STEC O113:H21 was centrifuged to separate bacteria and supernatant; FLAG-tagged LCN2 was detected in the supernatant. As shown in Fig. 5B, SubAB-induced FLAG-tagged normal-sized LCN2 (LCN2) was barely detectable in the supernatant compared to control or mt SubAB-treated cells. TM-induced unglycosylated LCN2 (LCN2**) was clearly detected. (Fig. 6A). The supernatants from control plasmid-transfected cells did not prevent STEC growth, even in the presence of SubAB or TM. However, STEC O113:H21 growth was suppressed with the supernatant containing normal-sized FLAG-tagged LCN2 from untreated or mt SubAB. However, the growth was not inhibited with TM- and wt SubAB-treated supernatant from FLAG-tagged LCN2-transfected cells (Fig. 6B). We next investigated the effect of purified recombinant human LCN2 (rLCN2) on STEC growth. As shown in Fig. 6C, the growth of all STEC O113:H21 strains (e.g., wild-type, *ΔsubAB*, *Δstx2*) was suppressed in the presence of rLCN2. We also investigated the effect of rLCN2 on STEC O113:H21-produced toxins; rLCN2 did not affect toxin transcription and production (Fig. 6D, Supplementary Fig. S6). These findings indicate that LCN2 can suppress STEC O113:H21 growth without promotion of toxin transcription. However, SubAB- or TM-induced low molecular weight LCN2 did not have bacteriostatic activity.

### SubAB induces mouse LCN2 in RAW264.7 cells and mouse intestinal cells

**Figure 7:**
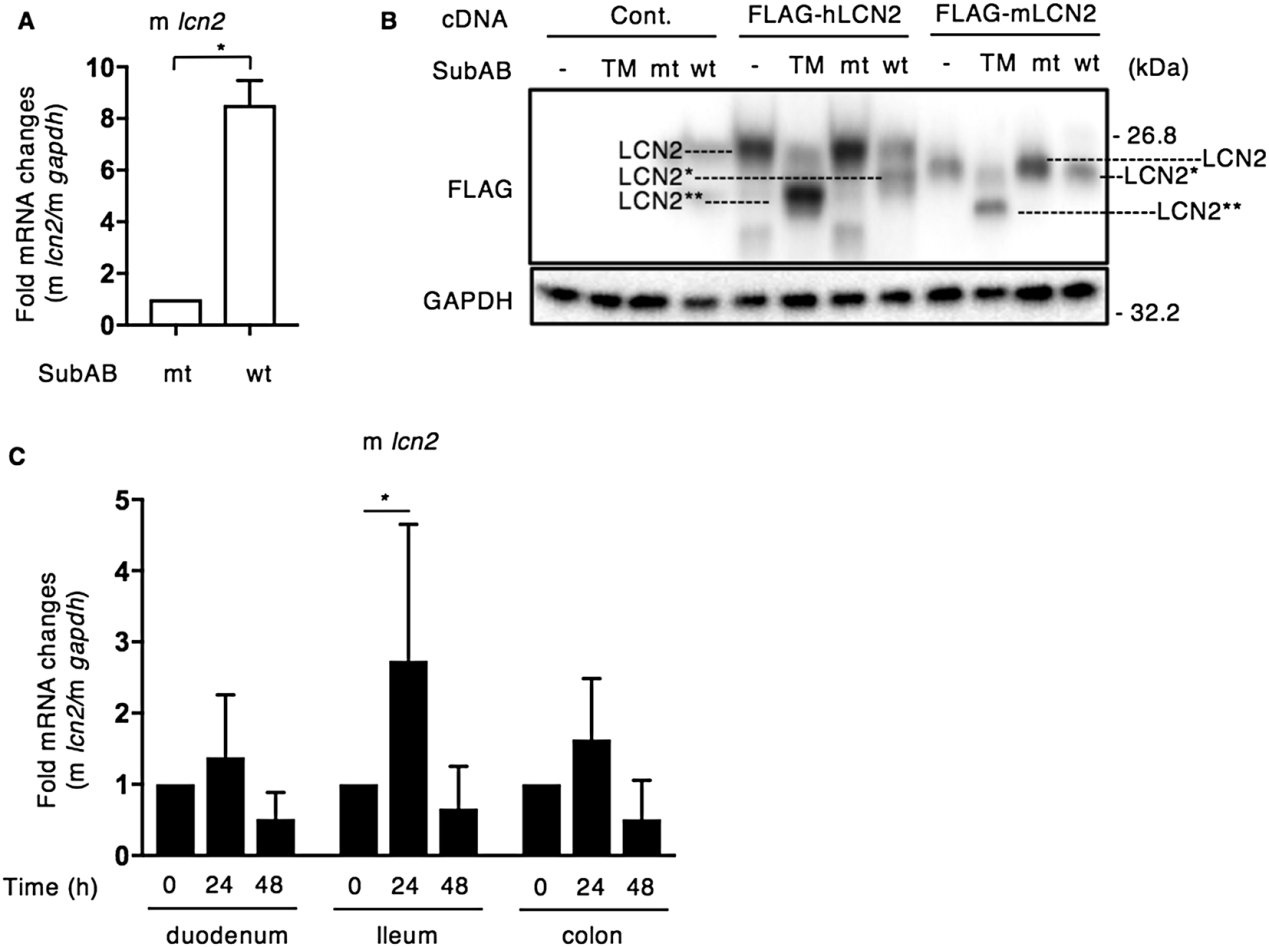
SubAB induces mouse LCN2 in RAW264.7 cells and mouse intestine. (**A**) RAW264.7 cells were incubated for 24 h with mt or wt SubAB. The mouse *lcn2* (m *lcn2*) mRNA levels were measured by RT-qPCR as described in “Methods”. GAPDH was used as an internal control. Data are mean ± SD (n = 3). *P < 0.05, versus mt SubAB-treated control cells. (**B**) HeLa cells were transfected with control, FLAG-hLCN2 or FLAG-mLCN2, and then incubated for 24 h with control DMSO, 1 μg ml^−1^ TM, 400 ng ml^−1^ mt SubAB or wt SubAB. Cell lysates were subjected to immunoblotting with the anti-FLAG antibodies. GAPDH served as a loading control. Experiments were repeated three times with similar results. (**C**) Mouse duodenum, ileum and colon were collected from treated mice at the indicated time points after the intraperitoneal injection of SubAB (10 μg/mouse) (n = 3 for each group). The m*lcn2* mRNA levels were measured by RT-qPCR as described in “Methods”. Mouse GAPDH (m *gapdh*) was used as an internal control. Data are mean ± SD (n = 3). *P < 0.05, versus mouse at 0 h.

The *lcn2* gene in the human genome exhibited only 62 percent similarity with the mouse orthologue^54^. To assess if mouse LCN2 (mLCN2) was also induced by SubAB, we investigated whether SubAB induces mLCN2 in RAW264.7 cells by RT-qPCR. SubAB-increased mouse *lcn2* (m *lcn2*) mRNA expression was observed after a 24 h incubation (Fig. 7A). We next cloned the mLCN2 gene from RAW264.7 cells and then constructed a FLAG-tagged mLCN2 expression vector. After HeLa cells were transfected with control, FLAG-tagged human LCN2 (hLCN2), or FLAG-tagged mLCN2 for 24 h, cells were treated for 24 h with TM, mt or wt SubAB. The molecular size of FLAG-tagged mLCN2 in control and in mt SubAB-treated cells was slightly lower compared to FLAG-tagged hLCN2. FLAG-tagged mLCN2 was also significantly shifted to low molecular weight by TM, and SubAB-caused a novel FLAG-tagged mLCN2 as well as FLAG-tagged hLCN2 (Fig. 7B).

We next investigated whether SubAB induces expression of *lcn2* mRNA in SubAB-injected mouse intraperitoneally. Mouse duodenum, ileum and colon were isolated after SubAB intraperitoneal injection for 0, 24 and 48 h, and then mRNA was purified. The mRNA level of mouse *lcn2* was measured by RT-qPCR. SubAB-stimulated mouse *lcn2* was increased in duodenum, ileum and colon after a 24 h injection (Fig. 7C).

## Discussion

Herein we found that STEC O113:H21-produced SubAB, not Stx2, increased LCN2 expression. SubAB-induced BiP cleavage activates PERK, and leads to phosphorylation of eukaryotic translation initiation factor 2α (eIF2α), followed by increased ATF4 and CHOP transcription^13,14^. CHOP plays an important role in ER stress-induced apoptosis^55^, with overexpression of CHOP causing cell cycle arrest and/or apoptosis^41^. In agreement, knockdown of CHOP reduced TM-induced apoptosis in HCC cells^56^. Overexpression of FLAG-tagged CHOP in HeLa cells enhanced SubAB-induced PARP cleavage after an 8 h incubation, not after a 24 h incubation. Interestingly, CHOP knockdown increased SubAB-induced PARP cleavage at 24 h. Consistent with our results, cigarette smoke extract-induced apoptosis was increased in CHOP-knockdown cells^57^. These findings indicate that the change in the amount of CHOP may be an important factor regulating apoptosis.

Knockdown of PERK and CHOP, but not ATF4, failed to induce LCN2, suggesting that the PERK/CHOP signaling pathway regulates LCN2 expression during ER stress. Analysis of LCN2 promoter region showed that the presence of transcriptional factor-binding sites (e.g., estrogen response element, NF-κB, C/EBPG, Vitamin D receptor, Stat1, Stat3, glucocorticoid response element). One previous study reported that LCN2 is a CHOP target gene, which mediates ER stress-induced apoptosis in A549 cells^40^. They suggested that thapsigargin-triggered ER stress-induced LCN2 required both CHOP and C/EBP binding to the LCN2 promoter. Heterodimer formation between CHOP and C/EBP proteins or activating transcription factor 4 (ATF4) is important in regulating transcriptional activity^58^. We could not detect SubAB-increased CHOP directly binding to LCN2 promoter by Chromatin Immunoprecipitation (ChIP) assay. Knockdown of C/EBPB and C/EBPG reduced SubAB-induced LCN2 and CHOP. LCN2 was not increased by CHOP overexpression alone. Taken together, our results suggest that the SubAB-increased C/EBPB or C/EBPG leads to CHOP increase, followed by formation of a complex with CHOP and C/EBP to stimulate LCN2 expression.

Previous study showed that tunicamycin (TM), *N*-glycosylation inhibitor and ER stress inducer, induced expression of an unglycosylated LCN2, which had no effect of its secretion into culture supernatant^48^. Consistent with the report, tunicamycin treatment induced shift of LCN2 to a smaller size (LCN2** in Fig. 5A), which was secreted into supernatant (Fig. 5B, right panels). SubAB-induced novel LCN2 was shifted on Western blots (LCN2* in Fig. 5A) with the size of LCN2* being slightly different from LCN2**. In the supernatant of SubAB-treated FLAG-LCN2 transfected cells, normal-sized LCN2 was decreased and LCN2* was difficult to detect (Fig. 5B), indicating that LCN2* in SubAB-treated cells might not be secreted due to abnormal post-translational modification. *N*-Glycosylation in eukaryotic organisms is generally mediated through a membrane protein complex located in the ER that transfers an oligosaccharide to individual Asn residues in Asn-X-(Ser/Thr) sequons^59^. From the amino acids sequence, human LCN2 possesses only one sequon (Asn85), suggesting that the difference of LCN2* and LCN2** was not due to the number of glycosylation sites. To investigate LCN2 glycosylation, we tested the effect of *N*-glycosidase F on FLAG-LCN2. FLAG-LCN2 overexpressing cells were incubated for 12 h with TM, or mt or wt SubAB, and then the cell lysates were treated with *N*-glycosidase F. As shown in Figure S5A, the FLAG-LCN2 from control- or mt SubAB-treated cells (LCN2) showed a significantly decreased molecular weight (LCN2**) by *N*-glycosidase F and was found at the same size as those seen in TM-treated cells. TM-induced LCN2** was not changed by *N*-glycosidase F. SubAB-stimulated LCN2* was slightly shifted down by the enzyme. We next looked at whether SubAB directly cleaves LCN2. SubAB was incubated for 2 h with rLCN2 or cell lysate at 37 °C. We detected LCN2 or BiP as a positive control. The molecular weight of LCN2 was not changed the molecular weight by SubAB. BiP (78-kDa) was not seen in the presence of SubAB (Supplementary Fig. S5B). Further, we confirmed that the *lcn2* mRNA sequence from mt and wt SubAB was the same. Thus, the carbohydrate structure of LCN2* induced by SubAB may affect secretion. SubAB induces various disruptions to cell pathways (e.g., protein synthesis inhibition, cell cycle arrest, apoptosis, stress granules formation)^14,15,19^. Thus, another possibility is that SubAB causes failure of the secretion system or exosome formation. These events might affect the secretion of LCN2*. In the case of SubAB-produced LEE-negative STEC infection, expression of normal form LCN2 is decreased in infected tissues because of the formation of a novel LCN2. Therefore, it might be difficult to detect as a biomarker or to have a significant antimicrobial effect.

Since LCN2 is an antimicrobial, siderophore-binding protein^29^, the prevention of LCN2 secretion by SubAB may promote the ability of STEC O113:H21 to survive in the host. On the other hand, the recombinant-human LCN2 treatment of STEC O113:H21 or O157:H7 Sakai strains could significantly suppress growth without induction of Stx2 or SubAB expression (Fig. 6, Supplementary Fig. S6). The gene for Stx is encoded on lambdoid bacteriophages integrated into the STEC genome, and phage induction is connected with the bacterial SOS response^60^. Thus, production of Stx2 in O157:H7 strain was stimulated by treatment with DNA-damaging agents, such as antibiotics^61–63^, H_2_O_2_^64^ or nitric oxide^65^, which is consistent with our result as shown in Supplementary Figure S6B. Thus, antimicrobial activity by LCN2 for STEC did not stimulate bacteriophage-mediated Stx2 production in STEC O157:H7 and O113:H21. Interestingly, we did not detect enhanced production of Stx2, which is encoded on bacteriophage integrated into the genome, or SubAB, by treatment of LEE-negative O113:H21 strain with H_2_O_2_ (Supplementary Fig. S6B).

Iron is an essential nutrient for all microorganisms and is used to catalyze various enzymatic reactions essential for growth^66^. LCN2 captures iron from microorganisms, thereby inhibiting their growth^25^. We investigated the effect of iron on STEC O113:H21 growth. As expected, the growth was promoted in an iron concentration-dependent manner (Supplementary Fig. S7A). Iron increased growth of O113:H21 strain, whereas a specific iron chelator 2,2′-dipyridyl (DIP), significantly suppressed growth. The growth was returned to control level in the presence of iron and DIP mixture (Supplementary Fig. S7B). Further, in the presence of ferric chloride, the levels of *subAB* and *stx2* mRNA were significantly increased (Supplementary Fig. S7C). These findings suggest that iron is an important factor for growth and toxin transcription in LEE-negative STEC O113:H21.

SubAB-induced apoptosis was promoted in LCN2-knockdown cells (Fig. 4B). LCN2 knockout by CRISPER/Cas9 plasmid decreased cell proliferation and increased sensitivity to cisplatin in prostate cancer cells^67^. LCN2-deficent mice showed increased intracellular labile iron, and were highly sensitive to LPS-induced mortality, associated with increased cell apoptosis and upregulation of proinflammatory gene expression^68^. Previous study suggested that decreased intracellular iron level induced cell death, which was prevented by delivery of iron^69^. SubAB-stimulated PARP cleavage was suppressed by addition of holo-Tf or FeCl_3_ (Fig. 5F), suggesting that intracellular iron level is an important factor for the SubAB-induced apoptosis pathway.

**Figure 8:**
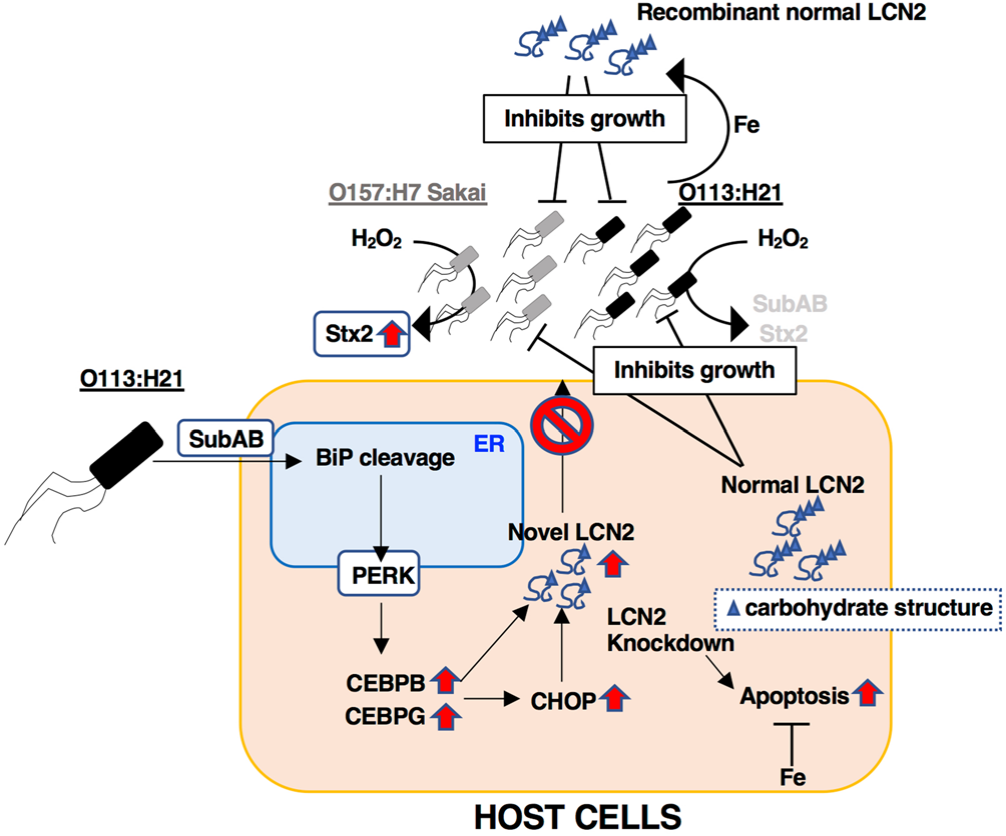
Proposed model of the signaling pathway by which SubAB induced novel LCN2 translation and host cell apoptosis. This figure is described in “Discussion”.

We proposed a model of this study as shown in Fig. 8. LEE-negative STEC O113:H21-produced SubAB is endocytosed into the ER, cleaves BiP, followed by activation of PERK. These events cause C/EBPB, and C/EBPG transcription, which further activates CHOP transcription. Overexpression of CHOP alone did not affect LCN2 expression and cell death. These transcriptional factors induce LCN2 transcription and translation of a novel-sized LCN2, which might not be secreted due to abnormal post-translational modification of carbohydrate structure and accumulated in cells. Increased intracellular iron level by holo-Tf or FeCl_3_ suppressed SubAB-induced PARP cleavage. However, over-expressed normal LCN2 and recombinant LCN2 prevented the siderophore iron-acquiring strategy of STEC, resulting in inhibition of STEC growth. SubAB-stimulated novel LCN2 did not show the inhibitory activity to growth of STEC. H_2_O_2_ treatment of LEE-positive STEC O157:H7 Sakai strain caused Stx2 expression, which was not stimulated by LCN2. In contrast, in LEE-negative STEC O113:H21 strain, the expression of toxins by treatment with LCN2 or H_2_O_2_ was not induced.

## Methods

### Subtilase cytotoxin preparation

*Escherichia coli *(*E. coli*) BL21 were used to produce recombinant His-tagged wild-type subtilase cytotoxin (wt SubAB) or catalytically inactivated mutant SubA (S272A)B (mt SubAB), which were purified using Ni-nitrilotriacetic acid (NTA) agarose (Qiagen) by published procedure^15^.

### Antibodies and other reagents

Antibodies against Lipocalin 2 (LCN2) (#44058), cleaved PARP (cPARP) (#5625), CHOP/GADD153 (#2895), PDI (#3501) and PERK (#3192) were purchased from Cell Signaling Technology. Mouse monoclonal antibodies against BiP/GRP78 (#610978) were from BD Biosciences. Anti-FLAG (#014–22383) monoclonal antibody, holo-transferrin (holo-Tf), Brefeldin A, and ferritic chloride (FeCl_3_) were from Fujifilm WAKO Pure Chemical Corporation. As an internal control of *E. coli*, *E. coli* RNA polymerase alpha (RNAPα) monoclonal antibody (#WP003) was obtained from BioLegend. Anti-GAPDH antibody was from GeneTex. Anti-SubAB and Stx2 antibodies were prepared as described previously^70^. 2,2′-Dipyridyl (DPI) and Deferoxamine mesylate salt (DF) were from Sigma Aldrich; and recombinant human LCN2 (1757-LC-050) was from R&D systems. *N*-Glycosidase F (P0704S) was purchased from New England BioLabs.

### Cell culture and gene transfection

HeLa and RAW264.7 cells (RIKEN cell bank) were cultured in RPMI1640 medium (Sigma) and HCT116 and Caco2 cells were cultured in D-MEM medium (Sigma) containing 10% fetal calf serum in the presence of Penicillin–Streptomycin Solution (Sigma). Cells were plated into 24-well dishes (5 × 10^4^ cells/well) or 12-well dishes (1 × 10^5^ cells/well) with medium containing 10% FCS. RNA interference-mediated gene knockdown was performed using validated Qiagen HP small-interfering RNAs (siRNAs) for PERK (SI02223718), C/EBPB (SI02777292), and C/EBPG (SI02777299). LCN2 siRNA (5′-gcaugcuaugguguucuuctt-3′)^71^ and ATF4 siRNA (5′-uucagugauauccacuucacugccc-3′)^72^ were designed and validated as described. CHOP siRNA was from Santa Cruz Biotechnology. Cells were transfected with 100 nM of the indicated siRNAs for 24–48 h using Lipofectamine RNAiMax transfection reagent (Invitrogen) according to the manufacturer’s protocol. Knockdown of the target proteins was confirmed by immunoblotting with the indicated antibodies.

### Construction of FLAG-tagged LCN2

Total mRNA was purified by ISOGEN II (NIPPON GENE) from SubAB-treated HeLa cells and RAW264.7 cells, and then cDNA was amplified using PrimeSTAR HS DNA Polymerase (TaKaRa Bio Inc., Japan) in a 25 μl PCR mixture according to the manufacturer’s protocol. The PCR conditions were as follows: 30 cycles of 98 ℃ for 15 s, 60 ℃ for 30 s, and 72 ℃ for 30 s. Primers used for PCR are shown in Supplementary Table S1. PCR products were ligated into the NotI and SalI sites of FLAG-5a expression vector (Sigma) using In-Fusion HD Cloning Kit (TaKaRa Bio Inc., Japan). The FLAG-tagged human or mouse LCN2 plasmid was sequenced and then transfected into HeLa cells with using Polyethylenimine Max (Polysciences) in OPTI-MEM I reduced-serum medium (ThermoFisher Scientific), according to the manufacturer’s protocol.

### Real-time quantitative reverse transcription polymerase chain reaction (RT-qPCR)

Total mRNA was purified by ISOGEN II (NIPPON GENE) from SubAB-treated cells, STEC strains, or intraperitoneal SubAB-injected mice, and then cDNA was amplified using PrimeSTAR HS DNA Polymerase (TaKaRa Bio Inc.) in a 20 μl PCR mixture according to the manufacturer’s protocol. Real-time quantitative PCR analysis was used with KOD SYBER qPCR Mix (TOYOBO) and ABI Prism 7000 (PerkinElmer Life Sciences). Primers used for PCR are shown in Supplementary Table S1. Relative expression was normalized to *gapdh* or *etuA* and calibrated to the respective controls^73^.

### STEC strains and growth conditions

STEC O157:H7 Sakai and O113:H21 were grown overnight in LB broth, which has 1.0–2.5 × 10^8^ cfu ml^−1^. The cultures were diluted at 10^–3^ or 10^–5^ in RPMI1640 medium and then grown at 37 °C, followed by addition of LCN2 or 0.1 mM H_2_O_2_. Bacterial growth was monitored at 595 nm optical density (OD_595_) using iMark Microplate reader (BioRAD) for the indicated time points at 37 ℃.

### Immunoblots analysis

The immunoblotting analysis was performed as described previously^22^. Briefly, proteins from cells were separated by SDS-PAGE and transferred to PVDF membranes, which were incubated with the indicated primary antibodies. Detection was performed with either horseradish peroxidase-labeled goat anti-mouse or anti-rabbit secondary antibodies (R&D systems), followed by enhanced chemiluminescence (EzWestLumi One, ATTO). Bands were visualized using Las 1000 (FUJIFILM). Densitometric analysis was performed by Image Gauge software (FUJIFILM) on the scanned blots, with proteins levels normalized to GAPDH.

### Immunofluorescence confocal microscopy

Immunofluorescence analysis of LCN2, CHOP and FLAG-tagged LCN2 was performed as described previously^19,74^. HeLa cells (2 × 10^5^ cells) on glass (Matsunami Glass Industries, Japan) were incubated with 400 ng ml^−1^ SubAB or mt SubAB for the indicated time. Cells were fixed with 4% paraformaldehyde (PFA) (Fujifilm WAKO Pure Chemical Corporation) for 30 min. The cells are then rinsed three times with PBS and incubated with blocking buffer (5% goat serum, 0.3% Triton X-100 in PBS) at room temperature for 1 h. To visualize FLAG, PDI, or CHOP, cells were further incubated with primary antibodies in 0.4% BSA/PBS buffer at 4 °C overnight, washed twice with PBS and incubated with anti-mouse 488 (Cell Signaling) or anti-rabbit Cy3 (Rockland) antibodies at room temperature for 1 h in the dark. After washing with PBS three times, cells were mounted on glass slides using Prolong Gold Antifade reagent with DAPI (Thermo Fisher Scientific). The stained cells were visualized by FV10i-LIV confocal microscopy (Olympus). The images were arranged with Adobe Photoshop CS4.

### Animal treatments

Male BALB/c mice (Japan SLC, Japan), 8 weeks old, were injected intraperitoneally with 10 µg/100 µl PBS of purified mt or wt SubAB, and mouse duodenum, ileum and colon were collected from mice at the indicated time points. As a control, mice received 100 µl of PBS. Total mRNA was purified by ISOGEN II (NIPPON GENE) from the tissues.

### Statistics

The *P* values for densitometric analysis and RT-qPCR assays were determined by Student’s *t* test with Graphpad Prism software (Graphpad, San Diego, CA, USA). *P* values of < 0.05 were considered statistically significant.

### Ethical approval

Animal experiments were approved by Chiba university committee for animal experiments. All experiments were performed in accordance with relevant guidelines and regulations.

## Supplementary information

Supplementary Information

## Abbreviations

SubAB: Subtilase cytotoxin
LEE: Locus of enterocyte effacement
STEC: Shiga-toxigenic *Escherichia coli*
LPS: Lipopolysaccharide
C/EBP: CCAAT enhancer binding protein
CHOP: C/EBP homology protein
PERK: RNA-dependent protein kinase-like ER kinase
TM: Tunicamycin
eIF2α: Eukaryotic translation initiation factor 2α
LCN2: Lipocalin 2
PARP: Poly (ADP ribose) polymerase
cPARP: Cleaved PARP
NC: Non-targeting control
TM: Tunicamycin
RT-qPCR: Real-time quantitative reverse transcription polymerase chain reaction

## References

[1] Scheutz F (2014). Taxonomy meets public health: The case of Shiga toxin-producing *Escherichia coli*. Microbiol. Spectr..

[2] Karmali MA (2004). Prospects for preventing serious systemic toxemic complications of Shiga toxin-producing *Escherichia coli* infections using Shiga toxin receptor analogues. J. Infect. Dis..

[3] Stalb S (2018). Pro-inflammatory capacity of *Escherichia coli* O104:H4 outbreak strain during colonization of intestinal epithelial cells from human and cattle. Int. J. Med. Microbiol..

[4] Carbonari CC (2019). An Stx-EAEC O59:NM[H19] strain isolated from a hemolytic uremic syndrome case in Argentina. Rev. Argent Microbiol..

[5] Bai X (2019). Molecular characterization and comparative genomics of clinical hybrid Shiga toxin-producing and enterotoxigenic *Escherichia coli* (STEC/ETEC) strains in Sweden. Sci. Rep..

[6] Gould LH (2013). Increased recognition of non-O157 Shiga toxin-producing *Escherichia coli* infections in the United States during 2000–2010: Epidemiologic features and comparison with *E. coli* O157 infections. Foodborne Pathog. Dis..

[7] Paton AW, Srimanote P, Talbot UM, Wang H, Paton JC (2004). A new family of potent AB(5) cytotoxins produced by Shiga toxigenic *Escherichia coli*. J Exp Med.

[8] Byres E (2008). Incorporation of a non-human glycan mediates human susceptibility to a bacterial toxin. Nature.

[9] Yahiro K (2011). Identification of subtilase cytotoxin (SubAB) receptors whose signaling, in association with SubAB-induced BiP cleavage, is responsible for apoptosis in HeLa cells. Infect. Immun..

[10] Yamaji T (2019). A CRISPR screen using subtilase cytotoxin identifies SLC39A9 as a glycan-regulating factor. iScience.

[11] Chong DC, Paton JC, Thorpe CM, Paton AW (2008). Clathrin-dependent trafficking of subtilase cytotoxin, a novel AB5 toxin that targets the endoplasmic reticulum chaperone BiP. Cell Microbiol..

[12] Nagasawa S (2014). Uptake of Shiga-toxigenic *Escherichia coli* SubAB by HeLa cells requires an actin- and lipid raft-dependent pathway. Cell Microbiol..

[13] Wolfson JJ (2008). Subtilase cytotoxin activates PERK, IRE1 and ATF6 endoplasmic reticulum stress-signalling pathways. Cell Microbiol..

[14] Yahiro K (2012). Regulation of subtilase cytotoxin-induced cell death by an RNA-dependent protein kinase-like endoplasmic reticulum kinase-dependent proteasome pathway in HeLa cells. Infect. Immun..

[15] Morinaga N (2007). Two distinct cytotoxic activities of subtilase cytotoxin produced by Shiga-toxigenic *Escherichia coli*. Infect. Immun..

[16] Morinaga N, Yahiro K, Matsuura G, Moss J, Noda M (2008). Subtilase cytotoxin, produced by Shiga-toxigenic *Escherichia coli*, transiently inhibits protein synthesis of Vero cells via degradation of BiP and induces cell cycle arrest at G1 by downregulation of cyclin D1. Cell. Microbiol..

[17] Yahiro K, Morinaga N, Moss J, Noda M (2010). Subtilase cytotoxin induces apoptosis in HeLa cells by mitochondrial permeabilization via activation of Bax/Bak, independent of C/EBF-homologue protein (CHOP), Ire1 alpha or JNK signaling. Microb. Pathog..

[18] Tsutsuki H (2012). Subtilase cytotoxin enhances *Escherichia coli* survival in macrophages by suppression of nitric oxide production through the inhibition of NF-kappa B activation. Infect. Immun..

[19] Tsutsuki H (2016). Subtilase cytotoxin produced by locus of enterocyte effacement-negative Shiga-toxigenic *Escherichia coli* induces stress granule formation. Cell Microbiol..

[20] Funk J (2015). Cytotoxic and apoptotic effects of recombinant subtilase cytotoxin variants of Shiga toxin-producing *Escherichia coli*. Infect. Immun..

[21] Yahiro K (2014). DAP1, a negative regulator of autophagy, controls SubAB-mediated apoptosis and autophagy. Infect. Immun..

[22] Yahiro K (2018). Mechanism of inhibition of Shiga-toxigenic *Escherichia coli* SubAB cytotoxicity by steroids and diacylglycerol analogues. Cell Death Discov..

[23] Cole JN, Nizet V (2016). Bacterial evasion of host antimicrobial peptide defenses. Microbiol. Spectr..

[24] Steckbeck JD, Deslouches B, Montelaro RC (2014). Antimicrobial peptides: New drugs for bad bugs?. Expert Opin. Biol. Ther..

[25] Goetz DH (2002). The neutrophil lipocalin NGAL is a bacteriostatic agent that interferes with siderophore-mediated iron acquisition. Mol. Cell.

[26] Asimakopoulou A, Weiskirchen S, Weiskirchen R (2016). Lipocalin 2 (LCN2) expression in hepatic malfunction and therapy. Front. Physiol..

[27] Bu DX (2006). Induction of neutrophil gelatinase-associated lipocalin in vascular injury via activation of nuclear factor-kappaB. Am. J. Pathol..

[28] Shen F, Hu Z, Goswami J, Gaffen SL (2006). Identification of common transcriptional regulatory elements in interleukin-17 target genes. J. Biol. Chem..

[29] Flo TH (2004). Lipocalin 2 mediates an innate immune response to bacterial infection by sequestrating iron. Nature.

[30] Berger T (2006). Lipocalin 2-deficient mice exhibit increased sensitivity to *Escherichia coli* infection but not to ischemia-reperfusion injury. Proc. Natl. Acad. Sci. USA.

[31] Chan YR (2009). Lipocalin 2 is required for pulmonary host defense against Klebsiella infection. J. Immunol..

[32] Cramer EP (2017). Lipocalin-2 from both myeloid cells and the epithelium combats *Klebsiella pneumoniae* lung infection in mice. Blood.

[33] Wu G (2014). Mechanism and clinical evidence of lipocalin-2 and adipocyte fatty acid-binding protein linking obesity and atherosclerosis. Diabetes Metab. Res. Rev..

[34] Oikonomou KA (2012). Neutrophil gelatinase-associated lipocalin (NGAL) in inflammatory bowel disease: Association with pathophysiology of inflammation, established markers, and disease activity. J. Gastroenterol..

[35] Moschen AR (2016). Lipocalin 2 protects from inflammation and tumorigenesis associated with gut microbiota alterations. Cell Host Microbe.

[36] Moschen AR, Adolph TE, Gerner RR, Wieser V, Tilg H (2017). Lipocalin-2: A master mediator of intestinal and metabolic inflammation. Trends Endocrinol. Metab..

[37] Wang G (2017). Lipocalin-2 promotes endoplasmic reticulum stress and proliferation by augmenting intracellular iron in human pulmonary arterial smooth muscle cells. Int. J. Biol. Sci..

[38] Mahadevan NR (2011). ER stress drives Lipocalin 2 upregulation in prostate cancer cells in an NF-kappaB-dependent manner. BMC Cancer.

[39] Oyadomari S, Mori M (2004). Roles of CHOP/GADD153 in endoplasmic reticulum stress. Cell Death Differ..

[40] Hsin IL (2012). Lipocalin 2, a new GADD153 target gene, as an apoptosis inducer of endoplasmic reticulum stress in lung cancer cells. Toxicol. Appl. Pharmacol..

[41] Li Y, Guo Y, Tang J, Jiang J, Chen Z (2014). New insights into the roles of CHOP-induced apoptosis in ER stress. Acta Biochim. Biophys. Sin. (Shanghai).

[42] Borkham-Kamphorst E, Van de Leur E, Haas U, Weiskirchen R (2019). Liver parenchymal cells lacking Lipocalin 2 (LCN2) are prone to endoplasmic reticulum stress and unfolded protein response. Cell Signal.

[43] Lee S (2007). A dual role of lipocalin 2 in the apoptosis and deramification of activated microglia. J. Immunol..

[44] Rahimi S (2019). CRISPR/Cas9-mediated knockout of Lcn2 effectively enhanced CDDP-induced apoptosis and reduced cell migration capacity of PC3 cells. Life Sci..

[45] Kang SS (2018). Lipocalin-2 protects the brain during inflammatory conditions. Mol. Psychiatry.

[46] Qiu S, Chen X, Pang Y, Zhang Z (2018). Lipocalin-2 protects against renal ischemia/reperfusion injury in mice through autophagy activation mediated by HIF1alpha and NF-kappab crosstalk. Biomed. Pharmacother..

[47] Guo H, Jin D, Chen X (2014). Lipocalin 2 is a regulator of macrophage polarization and NF-kappaB/STAT3 pathway activation. Mol. Endocrinol..

[48] Borkham-Kamphorst E, Van de Leur E, Meurer SK, Buhl EM, Weiskirchen R (2018). *N*-Glycosylation of lipocalin 2 is not required for secretion or exosome targeting. Front. Pharmacol..

[49] Zhao P, Elks CM, Stephens JM (2014). The induction of lipocalin-2 protein expression in vivo and in vitro. J. Biol. Chem..

[50] Parmar T (2018). Lipocalin 2 plays an important role in regulating inflammation in retinal degeneration. J. Immunol..

[51] Wilson BR, Bogdan AR, Miyazawa M, Hashimoto K, Tsuji Y (2016). Siderophores in iron metabolism: From mechanism to therapy potential. Trends Mol. Med..

[52] Fischbach MA (2006). The pathogen-associated iroA gene cluster mediates bacterial evasion of lipocalin 2. Proc. Natl. Acad. Sci. USA.

[53] Wang Q (2019). Lipocalin 2 protects against *Escherichia coli* infection by modulating neutrophil and macrophage function. Front. Immunol..

[54] Charkoftaki G (2019). Update on the human and mouse lipocalin (LCN) gene family, including evidence the mouse Mup cluster is result of an “evolutionary bloom”. Human Genom..

[55] Hu H, Tian M, Ding C, Yu S (2018). The C/EBP homologous protein (CHOP) transcription factor functions in endoplasmic reticulum stress-induced apoptosis and microbial infection. Front. Immunol..

[56] Lei Y (2017). CHOP favors endoplasmic reticulum stress-induced apoptosis in hepatocellular carcinoma cells via inhibition of autophagy. PLoS One.

[57] Huang C (2015). Activation of the UPR protects against cigarette smoke-induced RPE apoptosis through up-regulation of Nrf2. J. Biol. Chem..

[58] Su N, Kilberg MS (2008). C/EBP homology protein (CHOP) interacts with activating transcription factor 4 (ATF4) and negatively regulates the stress-dependent induction of the asparagine synthetase gene. J. Biol. Chem..

[59] Mellquist JL, Kasturi L, Spitalnik SL, Shakin-Eshleman SH (1998). The amino acid following an asn-X-Ser/Thr sequon is an important determinant of N-linked core glycosylation efficiency. Biochemistry.

[60] Waldor MK, Friedman DI (2005). Phage regulatory circuits and virulence gene expression. Curr. Opin. Microbiol..

[61] Grif K, Dierich MP, Karch H, Allerberger F (1998). Strain-specific differences in the amount of Shiga toxin released from enterohemorrhagic *Escherichia coli* O157 following exposure to subinhibitory concentrations of antimicrobial agents. Eur. J. Clin. Microbiol. Infect. Dis..

[62] McGannon CM, Fuller CA, Weiss AA (2010). Different classes of antibiotics differentially influence Shiga toxin production. Antimicrob. Agents Chemother..

[63] Zhang X (2000). Quinolone antibiotics induce Shiga toxin-encoding bacteriophages, toxin production, and death in mice. J. Infect. Dis..

[64] Wagner PL, Acheson DW, Waldor MK (2001). Human neutrophils and their products induce Shiga toxin production by enterohemorrhagic *Escherichia coli*. Infect. Immun..

[65] Ichimura K (2017). Nitric oxide-enhanced Shiga toxin production was regulated by Fur and RecA in enterohemorrhagic *Escherichia coli* O157. Microbiologyopen.

[66] Soares MP, Weiss G (2015). The Iron age of host-microbe interactions. EMBO Rep..

[67] Rahimi S (2019). CRISPR/Cas9-mediated knockout of Lcn2 effectively enhanced CDDP-induced apoptosis and reduced cell migration capacity of PC3 cells. Life Sci..

[68] Srinivasan G (2012). Lipocalin 2 deficiency dysregulates iron homeostasis and exacerbates endotoxin-induced sepsis. J. Immunol..

[69] Devireddy LR, Gazin C, Zhu X, Green MR (2005). A cell-surface receptor for lipocalin 24p3 selectively mediates apoptosis and iron uptake. Cell.

[70] Yahiro K (2006). Identification and characterization of receptors for vacuolating activity of subtilase cytotoxin. Mol. Microbiol..

[71] Kim SL (2017). Lipocalin 2 negatively regulates cell proliferation and epithelial to mesenchymal transition through changing metabolic gene expression in colorectal cancer. Cancer Sci..

[72] Igarashi T (2007). Clock and ATF4 transcription system regulates drug resistance in human cancer cell lines. Oncogene.

[73] Ichimura K (2017). Nitric oxide-enhanced Shiga toxin production was regulated by Fur and RecA in enterohemorrhagic *Escherichia coli* O157. Microbiologyopen.

[74] Nagasawa S (2014). Uptake of Shiga-toxigenic *Escherichia coli* SubAB by HeLa cells requires an actin-and lipid raft-dependent pathway. Cell. Microbiol..

