# Supplementary material for: Subtilase cytotoxin induces a novel form of Lipocalin 2, which promotes Shiga-toxigenic Escherichia coli survival
Source: Sci Rep. 2020 Nov 3;10:18943. doi: 10.1038/s41598-020-76027-z (PMC7609767; doi:10.1038/s41598-020-76027-z)

Supplementary Information

Subtilase cytotoxin induces a novel form of Lipocalin 2, which promotes Shiga-toxigenic *Escherichia coli* survival

Kinnosuke Yahiro[*1], Kohei Ogura [2], Yoshiyuki Goto [3,4,5], Sunao Iyoda[6], Tatsuya Kobayashi[7], Hiroki Takeuchi[8], Makoto Ohnishi[6], Joel Moss[9]

[1]Department of Molecular Infectiology, Graduate School of Medicine, Chiba University, Japan; [2]Advanced Health Care Science Research Unit, Institute for Frontier Science Initiative, Kanazawa University, Japan; [3]Division of Molecular Immunology, Medical Mycology Research Center, Chiba University, Chiba, Japan; [4]Division of Mucosal Symbiosis, International Research and Development Center for Mucosal Vaccines, Institute of Medical Science, The University of Tokyo, Tokyo 108-8639, Japan; [5]AMED-PRIME, Japan Agency for Medical Research and Development, Tokyo 100-0004, Japan; [6]Department of Bacteriology I, National Institute of Infectious Diseases, Shinjuku-ku, Tokyo 162-8640, Japan; [7]Reproductive Medicine, Graduate School of Medicine, Chiba University, Japan; [8]Clinical Laboratory, University of Tsukuba Hospital, Japan; [9]Pulmonary Branch, National Heart, Lung, and Blood Institute, National Institutes of Health, Bethesda, MD 20892-1590, USA

Figure legend

Figure S1. SubAB-induced CHOP increases LCN2 expression.

Control (NC), ATF4 or CHOP siRNA-transfected Caco2 or HCT116 cells were incubated for 24 h with 400 ng ml^{-1} of mt or wt SubAB. The *lcn2* mRNA levels were measured by RT-qPCR as described in Materials and Methods. GAPDH was used as an internal control. Data are mean ± SD (n=3). **P* < 0.05, versus mt SubAB-treated control cells.

Figure S2. Effect of various C/EBP siRNA on their mRNA and CHOP expression

A and B, Control (NC), C/EBPA, C/EBPB or C/EBPG siRNA-transfected HeLa cells were incubated for 24 h with 400 ng ml^{-1} of mt or wt SubAB. The mRNA levels of *cebp*A, *cebp*B, *cebp*G or *chop* were measured by RT-qPCR as described in Materials and Methods and Table.1. GAPDH was used as an internal control. Data are mean ± SD (n=3). **P* < 0.05, versus mt SubAB-treated control cells.

Figure S3. Localization of CHOP in SubAB-treated cells

The indicated cDNA-transfected cells were incubated for 24 h with mt or wt SubAB. Cells were fixed with 4% PFA and reacted with the anti-CHOP antibodies (red) and observed by confocal microscopy. Cell nuclei were stained by DAPI (cyan).

Figure S4. Localization of FLAG-tagged LCN2 in SubAB-treated cells

The indicated cDNA-transfected cells were incubated for 24 h with mt or wt SubAB. Cells were fixed with 4% PFA and reacted with the anti-protein disulfide isomerase (PDI) (red) or anti-FLAG antibodies (green) and observed by confocal microscopy. High magnification of Figure 5D. Cell nuclei were stained by DAPI (cyan).

Figure S5. Effect of N-Glycosidase F on FLAG-LCN2(A) and SubAB on rLCN2 (B).

A. FLAG-tagged LCN2-transfected cells were incubated for 12 h with DMSO (-), 1 μg/ml tunicamycin (TM), 400 ng/ml mt or wt SubAB. Cell lysate proteins were incubated for 1 h with N-Glycosidase F (N-Gly) at 37 °C according to the instruction manuals. Cell lysate proteins were analyzed by Western blotting using anti-FLAG antibodies and anti-GAPDH antibodies, as an internal control. The blots shown are representative of three independent experiments.

B. SubAB (1 μg/μl) was incubated for 2 h with rLCN2 (100 ng/μl) or HeLa cell lysate (100 μg/μl) at 37 °C, and then proteins were analyzed by Western blotting using anti-

LCN2, anti-BiP and anti-GAPDH antibodies. The blots shown are representative of three independent experiments.

Figure S6. Effect of recombinant human LCN2 or H_2O_2 on toxin production by STEC O157:H7 Sakai and O113:H21 strain. A, STEC O157:H7 Sakai and O113:H21 strains were grown in LB broth overnight, diluted with RPMI1640 medium (1~2.5x 10^3 cfu), stationary cultured for 2 h at 37°C, and then incubated with purified human recombinant LCN2 (rLCN2, 1.5 μg/100 μl in the medium). Bacterial growth was monitored by OD_{595} at 0, 4, 10 or 24 h. Data are mean ± SD (n=5). *$P < 0.05$, versus control. **B,** STEC O157:H7 Sakai and O113:H21 strains were grown in LB broth overnight, diluted with RPMI1640 medium (1~2.5x 10^3 cfu), stationary cultured for 2 h at 37°C, and then incubated for 24 h with 0.1 mM H_2O_2 or purified human recombinant LCN2 (1.5 μg/100 μl), and the proteins of the cultured STEC were analyzed by Western blotting using anti-Stx2 antiserum, anti-SubAB antibodies, and anti-RNAPα antibodies as an internal control. The blots shown are representative of three independent experiments.

Figure S7. Ferric chloride up-regulates STEC O113:H21 growth and toxin mRNA expression.

A, STEC O113:H21 strains were grown in LB broth overnight, diluted with RPMI1640 medium, stationary cultured for 24 h at 37℃ with 0, 1, or 10 μM $FeCl_3$, and then bacteria growth was monitored by taking OD_{595}. **B,** The STEC strains were grown in LB broth overnight, diluted with RPMI1640 medium, stationary cultured for 24 h at 37℃ with or without 10 μM $FeCl_3$ in the presence or absence of 0.5 mM DIP, and then bacterial growth was monitored by OD_{595}. **C,** After cultivation of STEC O113:H21 strain with the indicated concentration of $FeCl_3$ for 24 h, the levels of *subAB* and *stx2* mRNA were analyzed by RT-qPCR. EtufA was used as an internal control. Data are mean ± SD (n=3). *$P < 0.05$, versus untreated control bacteria.

Table 1. List of primers used in this paper.

Fig. S1

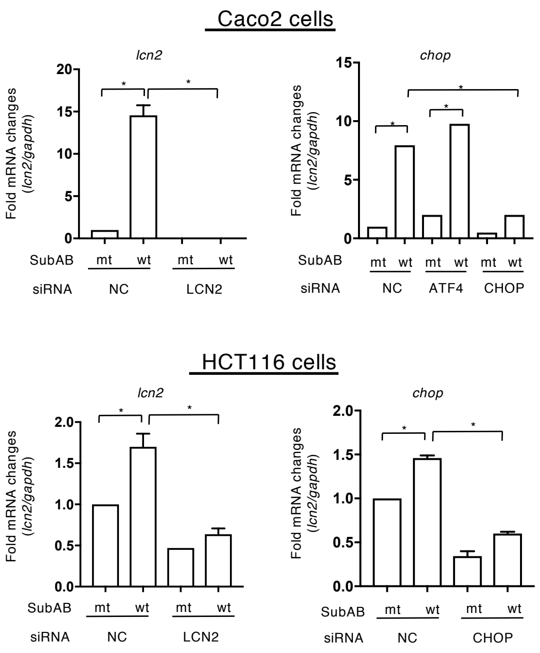
Caco2 cells
lcn2
Fold mRNA changes (lcn2/gapdh)
20
15
10
5
0
*
*
SubAB
mt
wt
mt
wt
siRNA
NC
LCN2
chop
Fold mRNA changes (lcn2/gapdh)
15
10
5
0
*
*
*
SubAB
mt
wt
mt
wt
mt
wt
siRNA
NC
ATF4
CHOP
HCT116 cells
lcn2
Fold mRNA changes (lcn2/gapdh)
2.0
1.5
1.0
0.5
0.0
*
*
SubAB
mt
wt
mt
wt
siRNA
NC
LCN2
chop
Fold mRNA changes (lcn2/gapdh)
2.0
1.5
1.0
0.5
0.0
*
*
SubAB
mt
wt
mt
wt
siRNA
NC
CHOP

Fig. S2

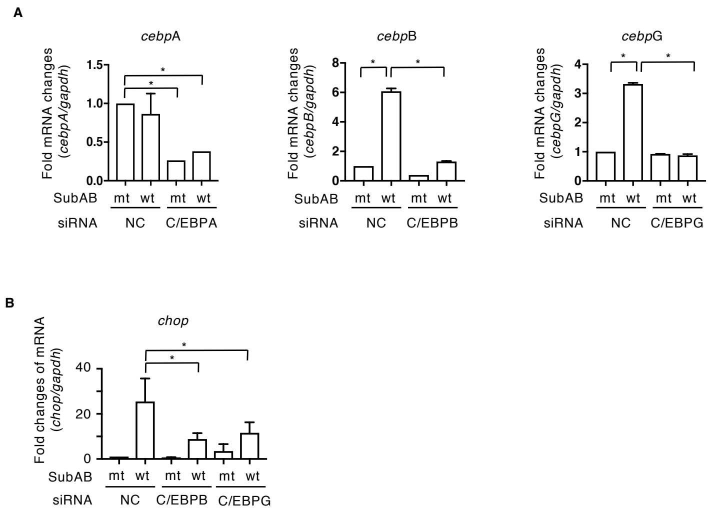
A
cebpA
Fold mRNA changes (cebpA/gapdh)
1.5
1.0
0.5
0.0
*
*
SubAB mt wt mt wt
siRNA NC C/EBPA
cebpB
Fold mRNA changes (cebpB/gapdh)
8
6
4
2
0
*
*
SubAB mt wt mt wt
siRNA NC C/EBPB
cebpG
Fold mRNA changes (cebpG/gapdh)
4
3
2
1
0
*
*
SubAB mt wt mt wt
siRNA NC C/EBPG
B
chop
Fold changes of mRNA (chop/gapdh)
40
20
0
*
*
SubAB mt wt mt wt mt wt
siRNA NC C/EBPB C/EBPG

Fig. S3

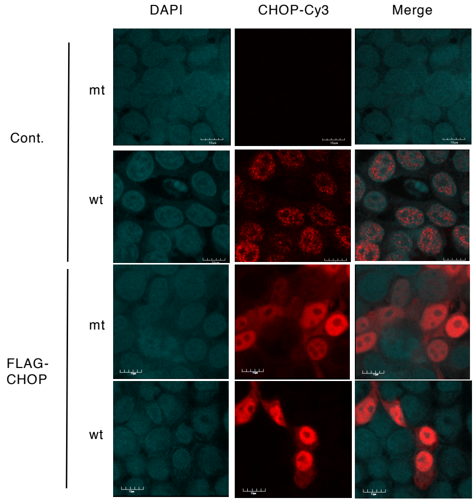

Fig. S4

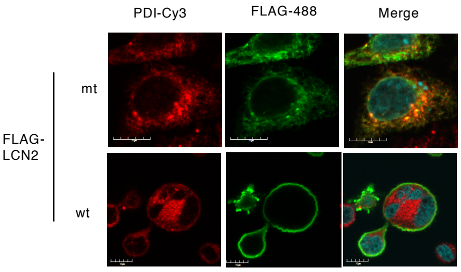

Fig. S5

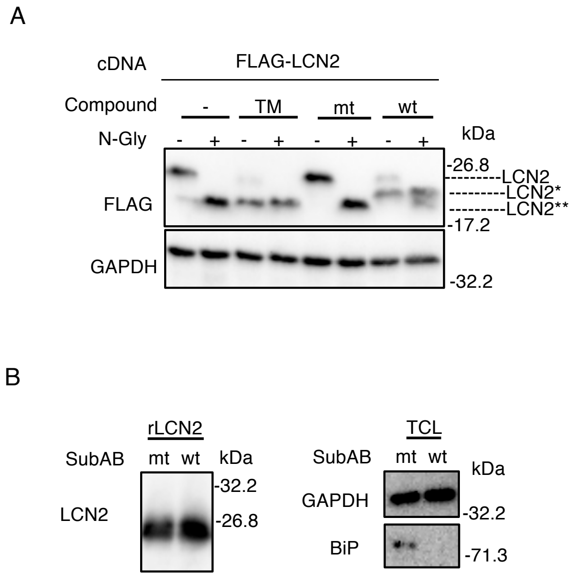
A
cDNA
FLAG-LCN2
Compound
-
TM
mt
wt
N-Gly
- + - + - + - +
kDa
FLAG
GAPDH
-26.8
LCN2
LCN2*
LCN2**
-17.2
-32.2
B
rLCN2
SubAB
mt wt
kDa
-32.2
LCN2
-26.8
TCL
SubAB
mt wt
kDa
GAPDH
-32.2
BiP
-71.3

Fig. S6

A

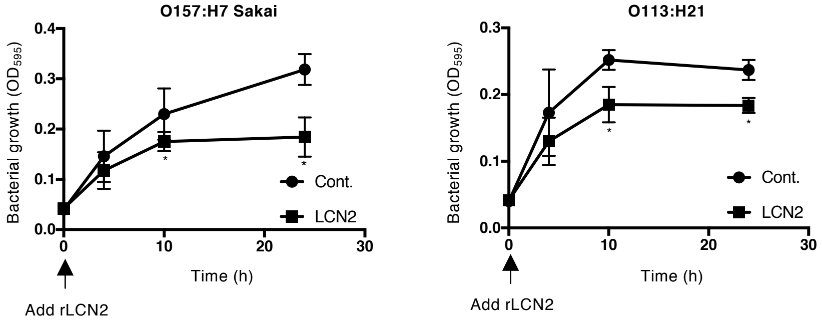
O157:H7 Sakai
Bacterial growth (OD_{595})
Time (h)
Cont.
LCN2
Add rLCN2
O113:H21
Bacterial growth (OD_{595})
Time (h)
Cont.
LCN2
Add rLCN2

B

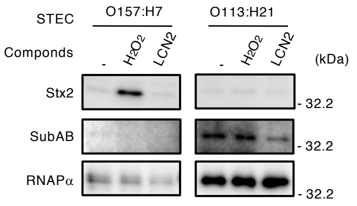
STEC
O157:H7
O113:H21
Componds
-
H_2O_2
LCN2
(kDa)
Stx2
SubAB
RNAPα
- 32.2

Fig. S7

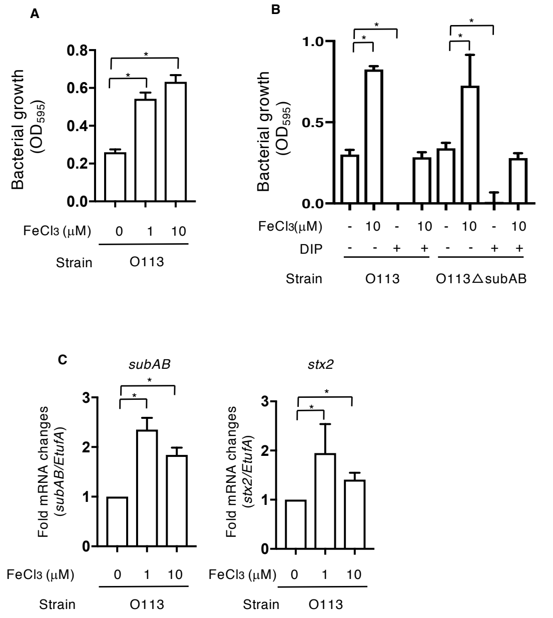
A
Bacterial growth (OD595)
0.0
0.2
0.4
0.6
0.8
FeCl3 (μM)
0
1
10
Strain
O113
B
Bacterial growth (OD595)
0.0
0.5
1.0
FeCl3(μM)
DIP
Strain
O113
O113△subAB
C
subAB
Fold mRNA changes (subAB/EtufA)
stx2
Fold mRNA changes (stx2/EtufA)
0
1
2
3

Table 1. List of oligonucleotides

Primers	Sequence
CHOP forward	5'-GGTATGAGGACCTGCAAGAGGT-3'
CHOP reverse	5'-CTTGTGACCTCTGCTGGTTCTG-3'
Human LCN2 forward	5'-GTGAGCACCAACTACAACCAGC-3'
Human LCN2 reverse	5'-GTTCCGAAGTCAGCTCCTTGGT-3'
mouse LCN2 forward	5'-ATGTCACCTCCATCCTGGTCAG-3'
mouse LCN2 reverse	5'-GCCACTTGCACATTGTAGCTCTG-3'
C/EBPA forward	5'-AACCTTGTGCCTTGGAAATG-3'
C/EBPA reverse	5'-GAGGCAGGAAACCTCCAAAT-3'
C/EBPB forward	5'-AGAAGACCGTGGACAAGCACAG-3'
C/EBPB reverse	5'-CTCCAGGACCTTGTGCTGCGT-3'
C/EBPG forward	5'-GCTTACAGCAGGTTCCTCAGCT-3'
C/EBPG reverse	5'-CGTTGCCGATACTCGTCACTGT-3'
Human GAPDH reverse	5'-GTCTCCTCTGACTTCAACAGCG-3'
Human GAPDH forward	5'-ACCACCCTGTTGCTGTAGCCAA-3'
mouse GAPDH reverse	5'-CATCACTGCCACCCAGAAGACTG-3'
mouse GAPDH forward	5'-ATGCCAGTGAGCTTCCCGTTCAG-3'
Stx2 forward	5'-CACATTTACAGTGAAGGTTGA3'
Stx2 reverse	5'-TTCAGCAAATCCGGAGCCTG-3'
subAB A subunit forward	5'-ACTCAGGATGGTTCACCGTT-3'
subAB A subunit reverse	5'-CCATGAGTCCTGTACACCGT-3'
EtufA forward	5'-TGGTTGATGACGAAGAGCTG-3'
EtufA reverse	5'-GCTCTGGTTCCGGAATGTAA-3'
Human FLAG-tagged LCN2 forward	5'-CAAGCTTGCGGCCGCCACCATGCCCC TAGGTCTCCTGTGGCT-3'
Human FLAG-tagged LCN2 reverse	5'-ACCGGATCCGTCGACGCCGTCGAT ACACTGGTCGA-3'
Mouse FLAG-tagged LCN2 forward	5'-CAAGCTTGCGGCCGCCACCATGGCCC TGAGTGTCATG -3'
Mouse FLAG-tagged LCN2 reverse	5'-ACCGGATCCGTCGACGTTGTCAATGC ATTGGTCGGTGGG -3'

Fig. 1B

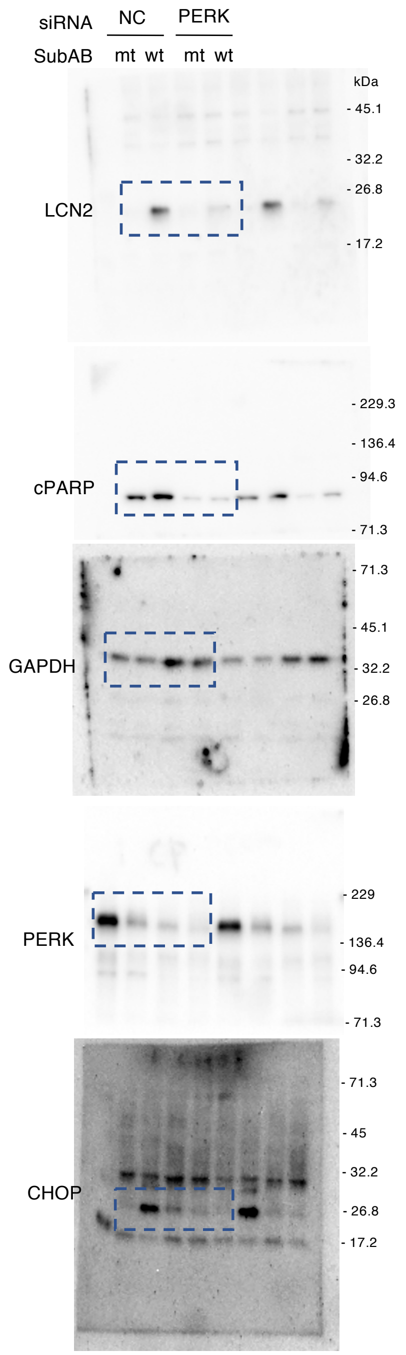

Fig. 1C

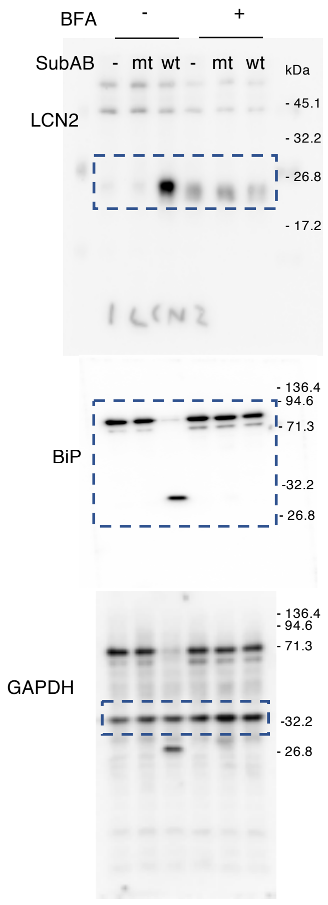

Fig. 1E

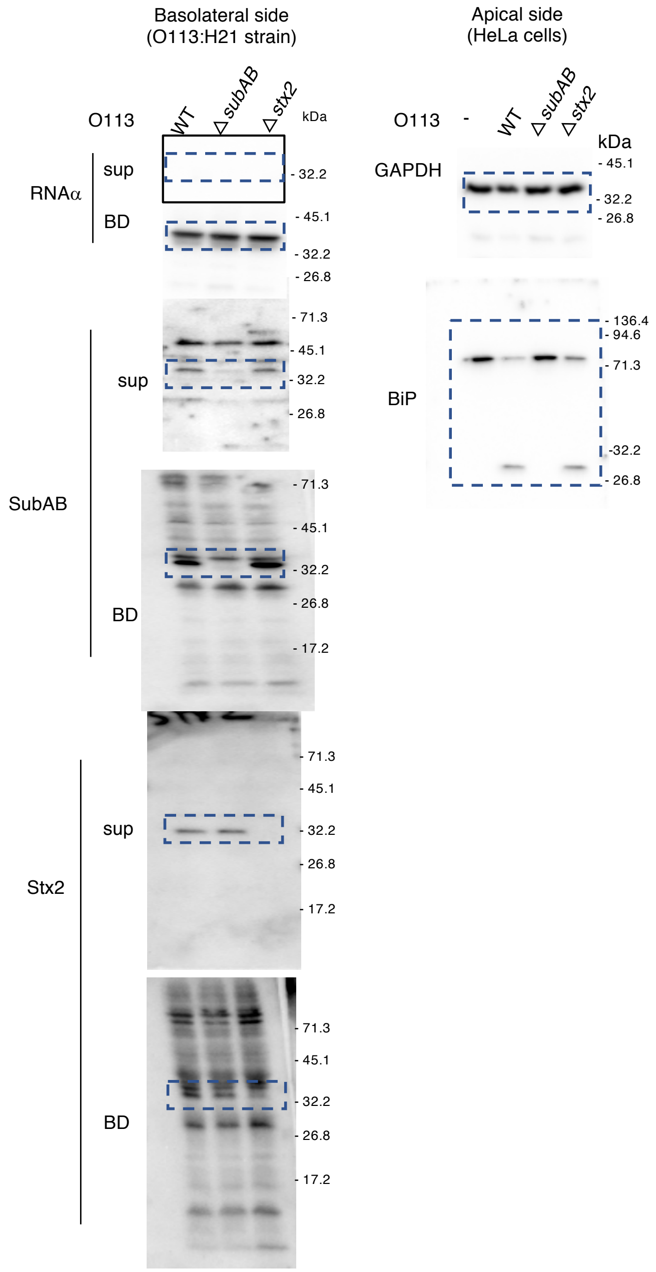

Fig. 2C

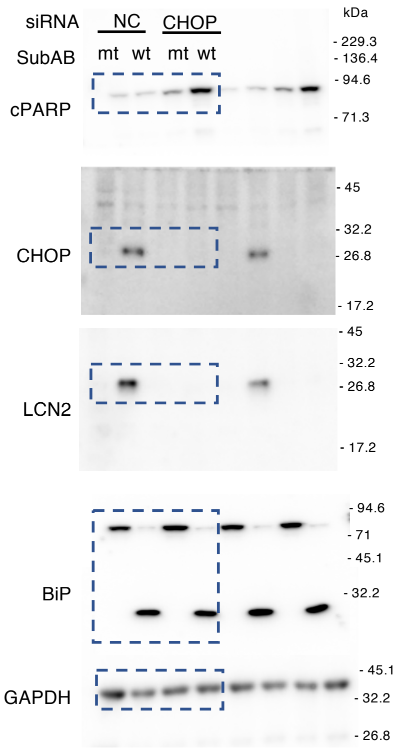

Fig. 2G

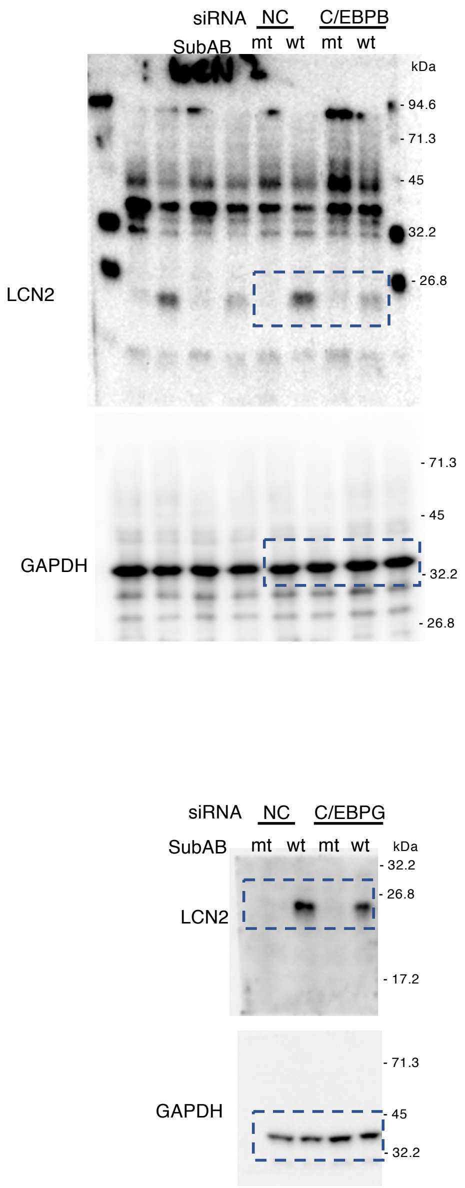
siRNA NC C/EBPB
SubAB mt wt mt wt
kDa
94.6
71.3
45
32.2
26.8
LCN2
71.3
45
GAPDH
32.2
26.8
siRNA NC C/EBPG
SubAB mt wt mt wt
kDa
32.2
26.8
LCN2
17.2
71.3
GAPDH
45
32.2

Fig. 3B

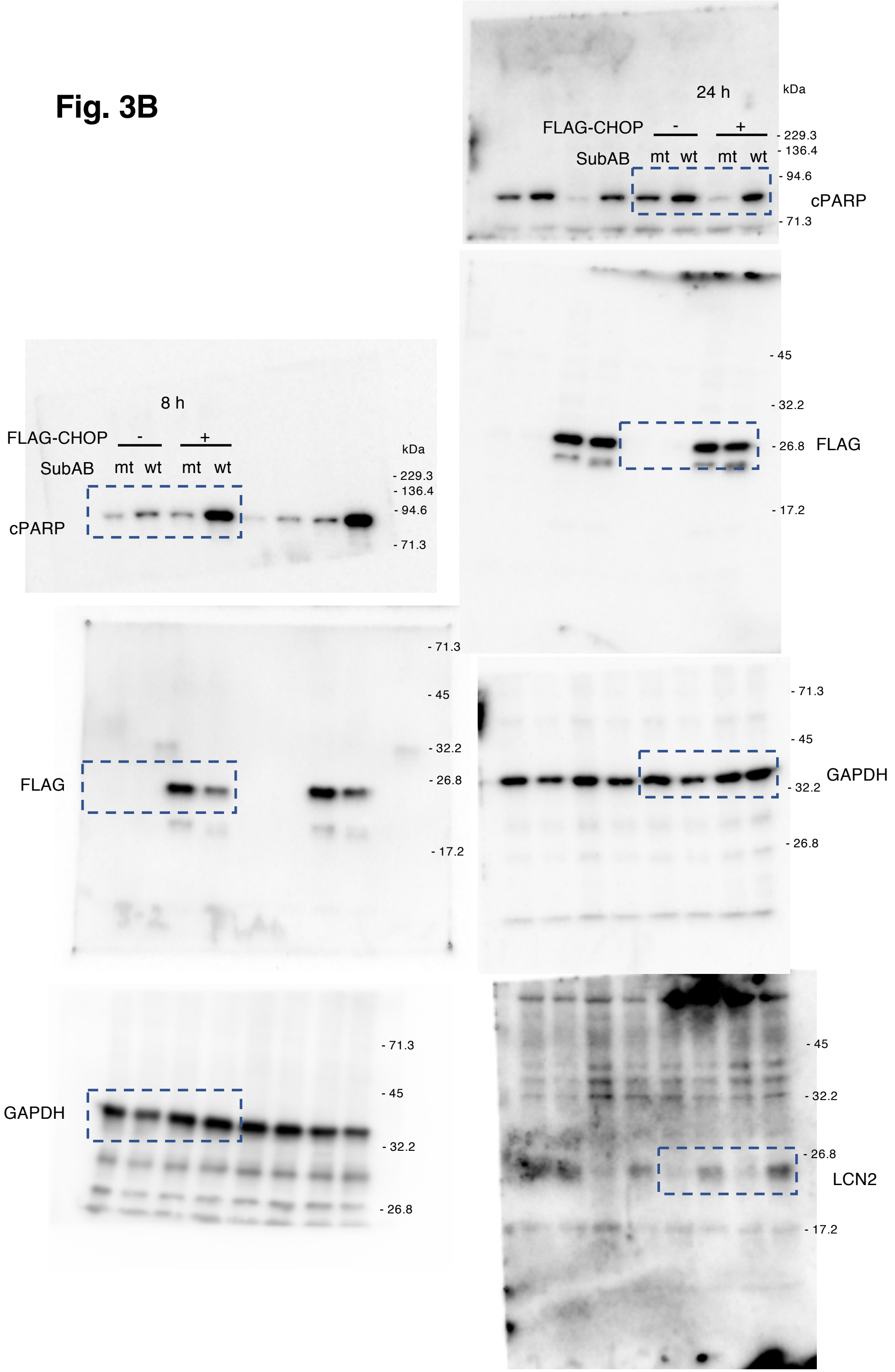
8 h
FLAG-CHOP - +
SubAB mt wt mt wt
kDa
229.3
136.4
94.6
cPARP
71.3
71.3
45
32.2
26.8
FLAG
17.2
71.3
45
GAPDH
32.2
26.8
24 h
kDa
FLAG-CHOP - +
229.3
136.4
SubAB mt wt mt wt
94.6
cPARP
71.3
45
32.2
26.8
FLAG
17.2
71.3
45
GAPDH
32.2
26.8
45
32.2
26.8
LCN2
17.2

Fig. 4B

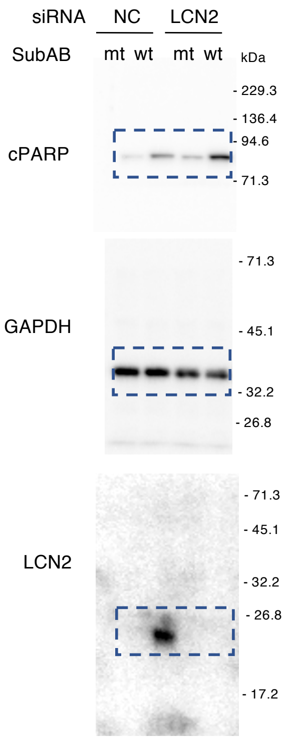
siRNA NC LCN2
SubAB mt wt mt wt
kDa
cPARP
- 229.3
- 136.4
- 94.6
- 71.3
GAPDH
- 71.3
- 45.1
- 32.2
- 26.8
LCN2
- 71.3
- 45.1
- 32.2
- 26.8
- 17.2

Fig. 4D

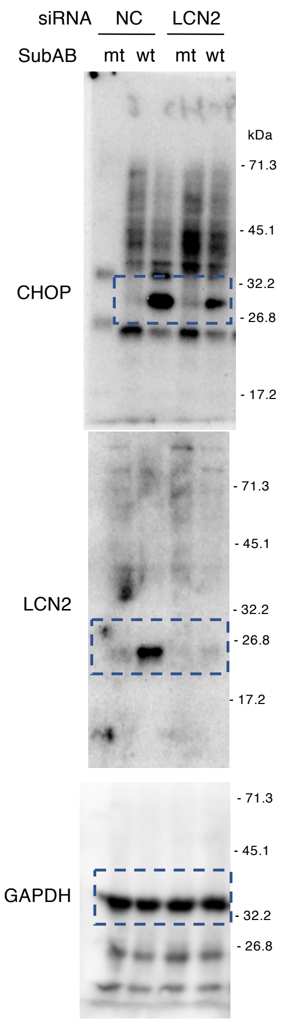
siRNA NC LCN2
SubAB mt wt mt wt
kDa
CHOP
- 71.3
- 45.1
- 32.2
- 26.8
- 17.2
LCN2
- 71.3
- 45.1
- 32.2
- 26.8
- 17.2
GAPDH
- 71.3
- 45.1
- 32.2
- 26.8

Fig. 5A

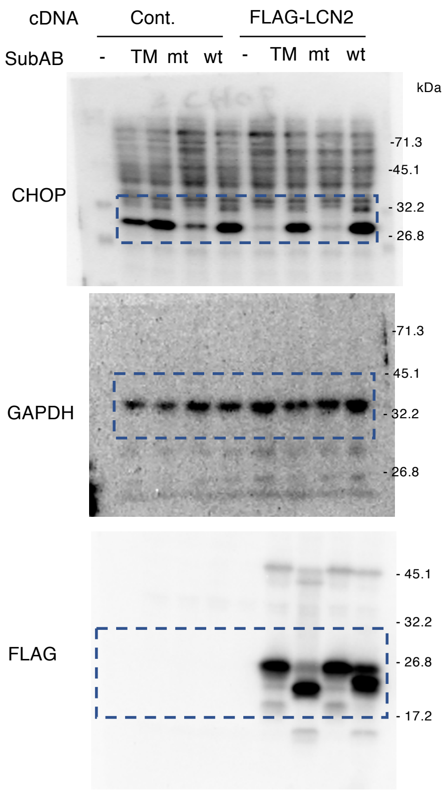

Fig. 5B

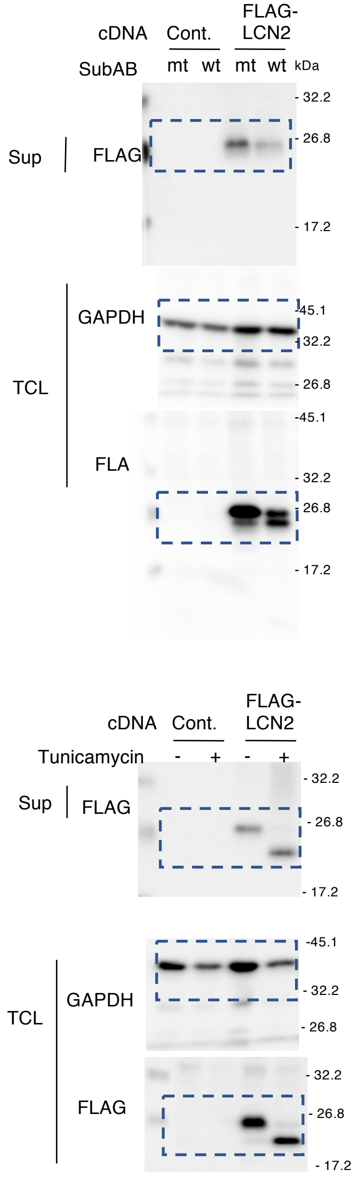

Fig. 5C

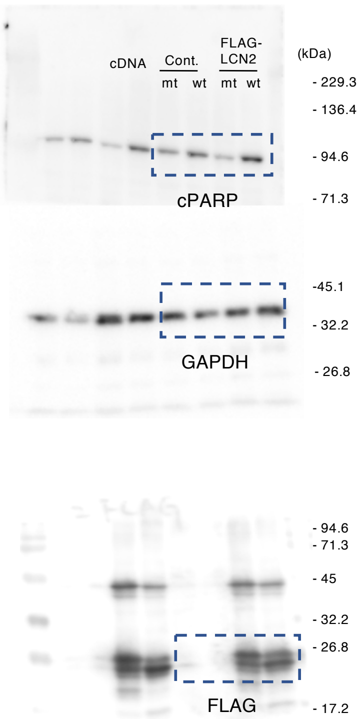

Fig. 5E

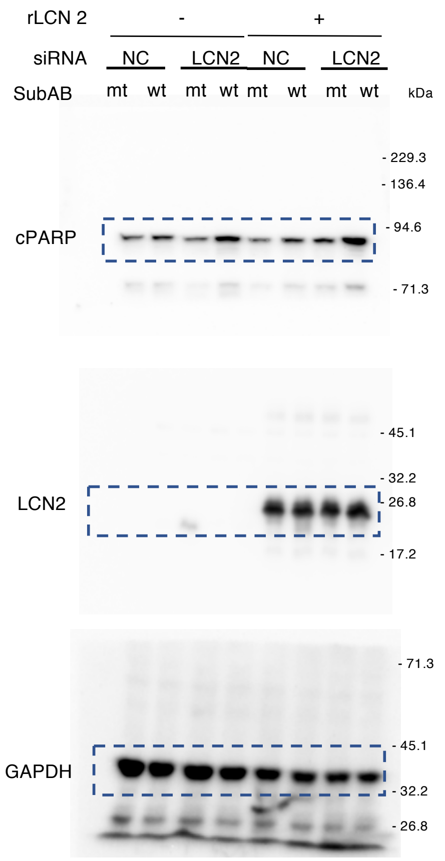

Fig. 5F

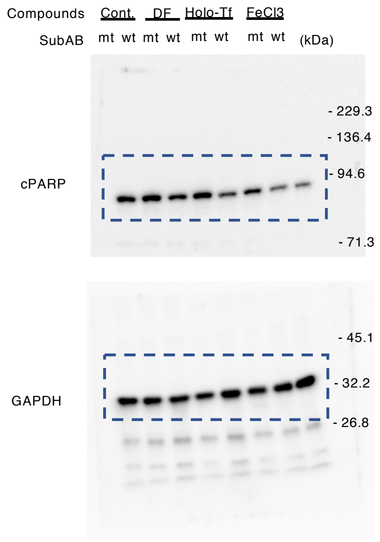

Fig. 7B

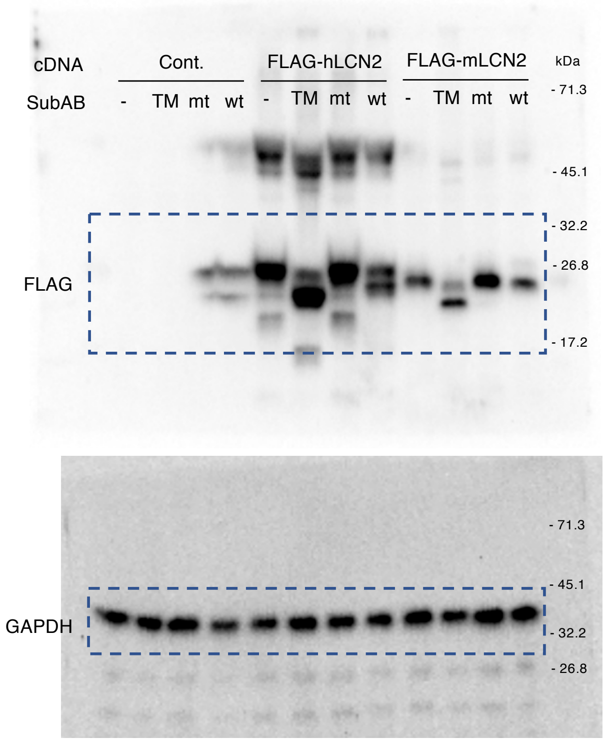

Fig. 6B

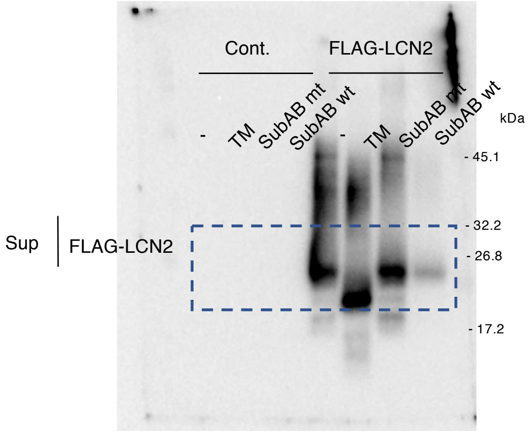

Fig. S5A

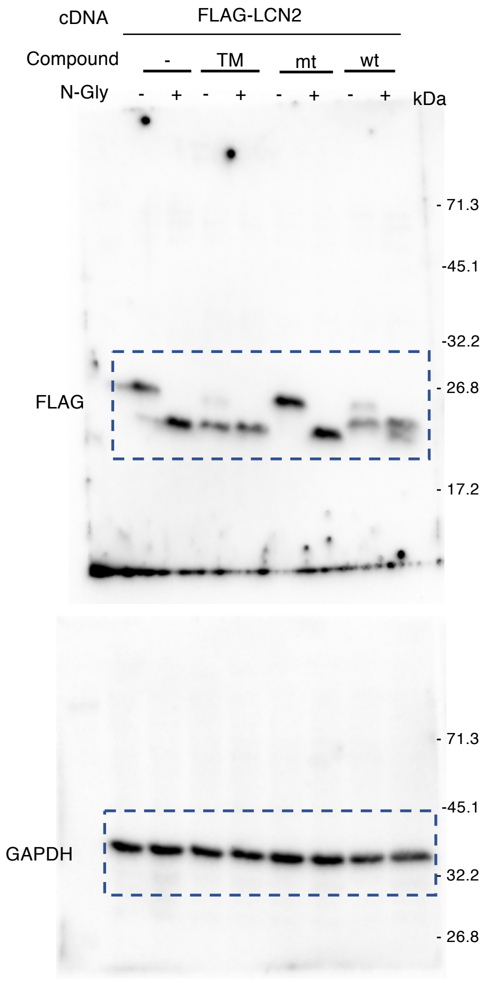

Fig. S5B

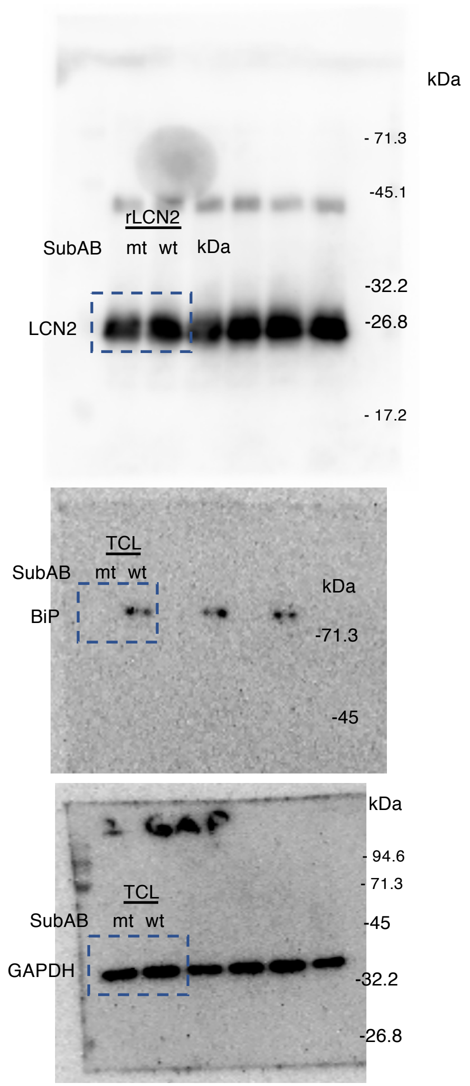

Fig. S6B

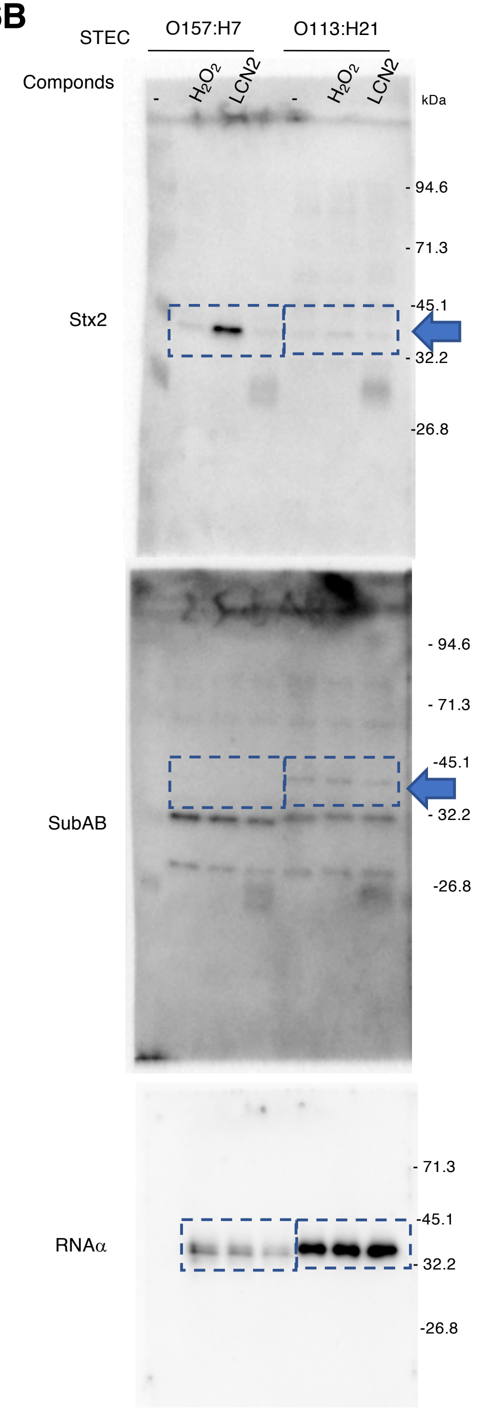

Supplement: Supplementary file 1 — Supplementary Information [file 41598_2020_76027_MOESM1_ESM.pdf]
